# Frailty and healthy working life expectancy: a multicohort modelling study

**DOI:** 10.7189/jogh.16.04338

**Published:** 2026-10-19

**Authors:** Yuhang Shui, Jingjie Zhu, Zeyu Huang, Huazhen You, Junling Gao

**Affiliations:** School of Public Health, Fudan University, Shanghai, China

**Keywords:** frailty, healthy working life expectancy, multistate Markov model, healthy ageing

## Abstract

**Background:**

Raising the retirement age and extending working years have become key strategies for many countries to mitigate the challenges of global population ageing. However, the association of frailty, a dynamic and reversible clinical condition, with healthy working life expectancy (HWLE) remains unclear.

**Methods:**

We used data from five harmonised health and retirement cohorts from across 33 countries, from which we tracked participants aged ≥50 years from baseline until their last available data point or death. Using this data, we evaluated frailty through the Frailty Index based on the cumulative deficit model, which classifies frailty status as robust, pre-frail, or frail. Then, we used discrete-time multistate Markov models to explore the association between frailty and HWLE, and further stratified our analyses by individual and country-level factors.

**Results:**

Among 180,314 participants, HWLE at the age of 50 years was 11.87 years (95% confidence interval (CI) = 11.74–12.00) for participants in the robust group, 7.34 years (95% CI = 7.19–7.50) for participants in the pre-frail group, and 3.77 years (95% CI = 3.57–3.97) for participants in the frail group. Compared with participants in the robust group, HWLE was 4.53 years shorter for those in the pre-frail group (Bonferroni-adjusted 95% confidence interval (95% CI_BA_) = −4.76, −4.29) and 8.11 years shorter for those in the frail group (95% CI_BA_ = −8.38, −7.84). Men had longer HWLE than women in the robust and pre-fail groups. Higher educational attainment and current non-smoking, as well as favourable national economic and political factors, were associated with longer HWLE across frailty strata.

**Conclusions:**

While frailty was generally associated with shorter HWLE at the age of 50 years, individual and country-level inequalities were also evident across frailty statuses. These results highlight a need to integrate targeted frailty prevention and intervention programmes with broader initiatives aimed at fostering national economic and political development, thereby promoting healthier, voluntary, and supported work participation in later life.

Global population ageing and the consequent increase in the number of retirees have led to a relative shortage in the labour force, which poses significant challenges to sustainable economic growth and development. Gradually raising the retirement age and extending working years have become crucial strategies for many countries attempting to mitigate this crisis [1–3]. Concurrently, promoting healthy longevity, rather than merely extending life span, has increasingly become a central objective within health systems. Given this context, the concept of healthy working life expectancy (HWLE) has been introduced to quantify the expected number of years for populations to remain within the workforce in a healthy state from the age of 50 years.

Frailty, defined as a nonspecific state of decreased physiological reserve, heightened vulnerability to stressors, and reduced homeostatic capacity in older adults, is associated not only with increased risks of adverse health outcomes and mortality [4–6], but also adverse employment outcomes such as unexpected retirement and unemployment [7], reduced working hours and income [8], and occupational falls [9,10]. 

From a life course perspective [11], an individual’s health and work status over time can be characterised as trajectories, which comprise transitions, *i.e.* changes between different states, and durations, *i.e.* the length of time spent in each state. Thus, we hypothesised that frailty may not only adversely affect subsequent health and work states, but may also be associated with unfavourable transitions between states and longer durations in unhealthy or not working states, thereby reducing the expected number of years spent both healthy and working.

Importantly, unlike disability, chronic diseases, and multimorbidity, which are often considered largely irreversible, frailty is a dynamic and reversible condition. Early identification and effective intervention, including tailored physical activity and nutritional programmes [12], may prevent adverse outcomes, such as declines in independence and functional capacity, thereby improving health human capital and potentially increasing HWLE at a population level. Yet notably, previous studies of HWLE have primarily focused on single countries and on disparities by gender, birth cohort, educational attainment, lifestyle, and chronic disease [13–18].

To our knowledge, evidence on the association between frailty and HWLE remains limited, with few studies having explored such associations from a multinational perspective. As a composite indicator that integrates both health and social policy dimensions, HWLE is shaped not only by one’s health status, but also by retirement policies and pension systems, labour market conditions, disability support, and economic and sociocultural circumstances. Therefore, understanding the potential heterogeneity in HWLE among populations with varying frailty statuses, along with underlying individual and country-level inequalities, is crucial for developing more equitable policies for extending working life and promoting the health development of older adults and society as a whole. Using 5 prospective ageing cohorts across 33 countries, we estimated HWLE at age 50 years by frailty statuses and further stratified the analyses by individual and country-level characteristics to explore which factors are associated with prolonged HWLE within specific frailty statuses and across national contexts.

## METHODS

We used data from 5 harmonised cohorts within the Gateway to Global Aging Data [19]: the Health and Retirement Study (HRS) 1992–2022, from which we used waves 7–16 out of 16 in total; the English Longitudinal Study of Ageing (ELSA), 2002–2019, from which we used waves 1, 2, and 4–6 out of 9 in total; the Survey of Health, Ageing and Retirement in Europe (SHARE), 2004–2022, from which we used waves 1, 2, and 4–9 from 9 in total; the Mexican Health and Aging Study (MHAS), 2001–2021, from which we used all 6 waves; and the China Health and Retirement Longitudinal Study (CHARLS), 2011–2018, from which we used all 4 waves. The Global Aging Data have been specifically harmonised to allow comparability of the self-reported measures from each cohort [20]. We report our findings per the STROBE reporting guidelines (Table S1 in the **Online Supplementary Document**) [21] and confirm the adherence of our secondary data analysis to the Journal of Global Health’s GRABDROP guidelines (Table S2 in the **Online Supplementary Document**) [22].

### Variables

#### Outcomes

We used self-rated health (SRH) as a measure of health, which serves as an effective indicator for tracking dynamic transitions in the health status of middle-aged and older adults, and has been widely used in previous calculations of healthy life expectancy [23–25] (Table S3 in the **Online Supplementary Document**). The measure was consistently available across cohorts and avoided overlap with functional deficits already included in the frailty index. We categorised responses of excellent, very good, or good as ‘good’, and responses of fair, poor, or very poor as ‘poor’.

We defined work states as participation in paid work or self-employment within the year of the interview, which we dichotomised into ‘working’ and ‘not working’ (Table S4 in the **Online Supplementary Document**). Death was identified through the respondent status recorded in each wave. We classified participants into one of five states: healthy and not working, healthy and working, not healthy and not working, not healthy and working, or deceased (Figure S1 in the **Online Supplementary Document**). Annual transition probabilities among these states at the age of 50 years by frailty were reported in Figure S2 in the **Online Supplementary Document**.

#### Age

We calculated the age at survival/death by subtracting birth month and year from the survey/death month and year. The median month was assigned if the birth month was missing; when a respondent was confirmed deceased while the specific death month and year were missing, the date of death was extrapolated from the closest interview date of the last wave and known vital status at this interview and was determined as the mid of the time interval between two consecutive interviews [16,26–28].

#### Exposures of interest

We calculated the Frailty Index (FI) as the reflection of frailty status based on the cumulative deficit model [29,30], with the construction aligning with previously set standards [31] (Figure S3, Tables S5 and S6, and Appendix S1 in the **Online Supplementary Document**). In detail, we coded selected health deficits from 0 to 1, after which we calculated the FI by dividing the sum of deficit scores by the total number of deficit items, and further categorising individuals into robust (FI score ≤0.10), pre-frail (FI score >0.10 to <0.25), or frail groups (FI score ≥0.25) [4]. Frailty status could change over time across waves.

#### Subpopulation identifiers

We classified our sample into subgroups by individual-level factors, *i.e.* gender, educational attainment, marital status, and current smoking and country-level factors, *i.e.* gross domestic product (GDP) *per capita*, Gini index, current health expenditure, tax revenue, civil society participation index (CSPI), government effectiveness, control of corruption, and political stability and absence of violence/terrorism (PSAVT), which were lagged by one year (Appendix S2 in the **Online Supplementary Document**). Country-level factors were dichotomised at the median to examine whether populations living in countries above or below median levels of economic and political indicators differed in HWLE across frailty statuses, and to facilitate interpretation of the results in policy-relevant terms [28,32,33]. Data on country-level indicators were obtained from the World Bank Group [34] and the V-Dem Institute [35].

### Statistical analysis

We used discrete-time multistate Markov models to analyse HWLE at the age of 50 years. Through biennial transition probability models with a 24-month interpolation step, participants were followed up approximately every two years. We estimated HWLE by frailty status and further stratified analyses by individual and country-level factors (Appendix S3 and Table S7 in the **Online Supplementary Document**). Missing country-level values were imputed using structural time series models with Kalman smoothing [36].

We tested differences in healthy working life expectancy (DHWLE) between subgroups by calculating the difference and the square root of the sum of variances of the two groups under the assumption of independence. We applied a Bonferroni correction to 95% confidence intervals (CIs), with a DHWLE considered statistically significant when the CI did not contain the null value. Significance levels of two-tailed tests were set to 0.05. We then conducted several sensitivity analyses. First, we further used both longer (with the step size 30 months) and shorter (with the step size 12 and 18 months) interpolations to reanalyse HWLE by frailty. Second, we reanalysed HWLE by frailty using the sample with complete individuals disregarding the missing information on subpopulation identifiers. We also applied inverse probability weighting based on gender, age, and cohort (Appendix S4 in the **Online Supplementary Document**) to reanalyse the association between frailty and HWLE, and thus assess the potential impact of selection bias on our findings. Third, we randomly resampled 70%, 80%, and 90% of the samples from our primary dataset to re-estimate HWLE by frailty. Fourth, we reclassified frailty status through two differential cutoffs on frailty, with robust status identified if FI was under 0.08 for the one cutoff and frail status identified if FI exceeded or was equal to 0.21 for the other compared to the original cutoff standard in the main analysis. Fifth, we used alternative imputation methods (linear, spline, and Stineman interpolation, and nearest neighbours with the last observation carried forward) to fill country-level missing values repeatedly and validate the robustness of our results under different methodological approaches. Finally, we estimated the association between frailty and HWLE stratified by baseline work state. We also presented the association between baseline frailty status and HWLE to reduce the potential influence of reverse causality [37].

We conducted all analyses in IMaCh, version 0.99s12 (*Institut national d'études démographiques*, Paris, France), *R*, version 4.5.1 (R Foundation for Statistical Computing, Vienna, Austria), and Stata, version 14.0 (StataCorp LLC, College Station, Texas, USA).

## RESULTS

The final sample comprised 180,314 participants: 29,398 from the HRS, 12,220 from the ELSA, 104,132 from the SHARE, 21 017 from the MHAS, and 13,547 from the CHARLS (**Table 1**; Figure S4 and Table S8 in the **Online Supplementary Document**). The mean age of our participants was 64.2 years (standard deviation (SD) = 10.1) years and 82,703 (45.9%) were men. Most participants were married (132,768, 73.6%), had low educational attainment (84,815, 47.0%), and were not current smokers (145,713, 80.8%). The average (SD) of follow-up was 8.19 years (SD = 5.19). Furthermore, 81,072 participants (45.0%) lived in countries with high GDP *per capita*, 68,440 (38.0%) in countries with low Gini index, 80,355 (44.6%) in countries with high current health expenditure, and 65,590 (36.4%) in countries with high tax revenue. A high CSPI, government effectiveness, control of corruption, and PSAVT were observed for 86,760 (48.1%), 88,158 (48.9%), 84,565 (46.9%), and 76,053 (42.2%) participants, respectively. At baseline, 81,919 participants (45.5%) were included in the robust group, 63,717 (35.3%) in the pre-frail group, and 34 678 (19.2%) in the frail group.

**Table 1:** Characteristics of participants at baseline

	Overall (n = 180,314)	HRS (n = 29,398)	ELSA (n = 12,220)	SHARE (n = 104,132)	MHAS (n = 21,017)	CHARLS (n = 13,547)
**Frailty status**						
Robust	81,919 (45.5)	8,658 (29.5)	6,316 (51.7)	53,188 (51.1)	8,527 (40.5)	5,230 (38.6)
Pre-frail	63,717 (35.3)	11,030 (37.5)	3,706 (30.3)	35,833 (34.4)	7,472 (35.6)	5,676 (41.9)
Frail	34,678 (19.2)	9710 (33.0)	2,198 (18.0)	15,111 (14.5)	5,018 (23.9)	2,641 (19.5)
**Age in years, x̄ (SD)**	64.23 (10.11)	64.01 (10.95)	64.47 (9.91)	65.26 (10.12)	60.34 (8.73)	62.61 (8.45)
**Gender**						
Women	97,611 (54.1)	16,394 (55.8)	6,395 (52.3)	56,697 (54.4)	11,157 (53.1)	6,968 (51.4)
Men	82,703 (45.9)	13,004 (44.2)	5,825 (47.7)	47,435 (45.6)	9,860 (46.9)	6,579 (48.6)
**Educational attainment**						
Low	84,815 (47.0)	6,325 (21.5)	5,027 (41.1)	43,453 (41.7)	17,877 (85.1)	12,133 (89.5)
Intermediate	63,099 (35.0)	16,893 (57.5)	5,398 (44.2)	39,066 (37.5)	553 (2.6)	1,189 (8.8)
High	32,400 (18.0)	6,180 (21.0)	1,795 (14.7)	21,613 (20.8)	2,587 (12.3)	225 (1.7)
**Marital status**						
Unmarried	47,546 (26.4)	10,252 (34.9)	3,610 (29.5)	26,107 (25.1)	5,539 (26.4)	2,038 (15.0)
Married	132,768 (73.6)	19,146 (65.1)	8,610 (70.5)	78,025 (74.9)	15,478 (73.6)	11,509 (85.0)
**Current smoking**						
No	145,713 (80.8)	24,169 (82.2)	10,049 (82.2)	84,540 (81.2)	17,541 (83.5)	9,414 (69.5)
Yes	34,601 (19.2)	5,229 (17.8)	2,171 (17.8)	19,592 (18.8)	3,476 (16.5)	4,133 (30.5)

CHARLS – the China Health and Retirement Longitudinal Study, ELSA – the English Longitudinal Study of Ageing, HRS – the Health and Retirement Study, MHAS – the Mexican Health and Aging Study, SD – standard deviation, SHARE – the Survey of Health, Ageing and Retirement in Europe, x̄ – mean

*Presented as n (%) unless specified otherwise.

### HWLE by frailty

The HWLE at the age of 50 years was 9.64 years (95% CI = 9.52–9.76) among the total population, 11.87 years (95% CI = 11.74–12.00) in the robust group, 7.34 years (95% CI = 7.19–7.50) in the pre-frail group, and 3.77 years (95% CI = 3.57–3.97) in the frail group (**Figure 1**; Figure S5 and Table S9 in the **Online Supplementary Document**). Compared with the robust group, HWLE was 4.53 years shorter for the pre-frail group (Bonferroni-adjusted 95% confidence interval (95% CI_BA_) = −4.76, −4.29) and 8.11 years shorter for the frail group (95% CI_BA_ = −8.38, −7.84).

**Figure 1:**
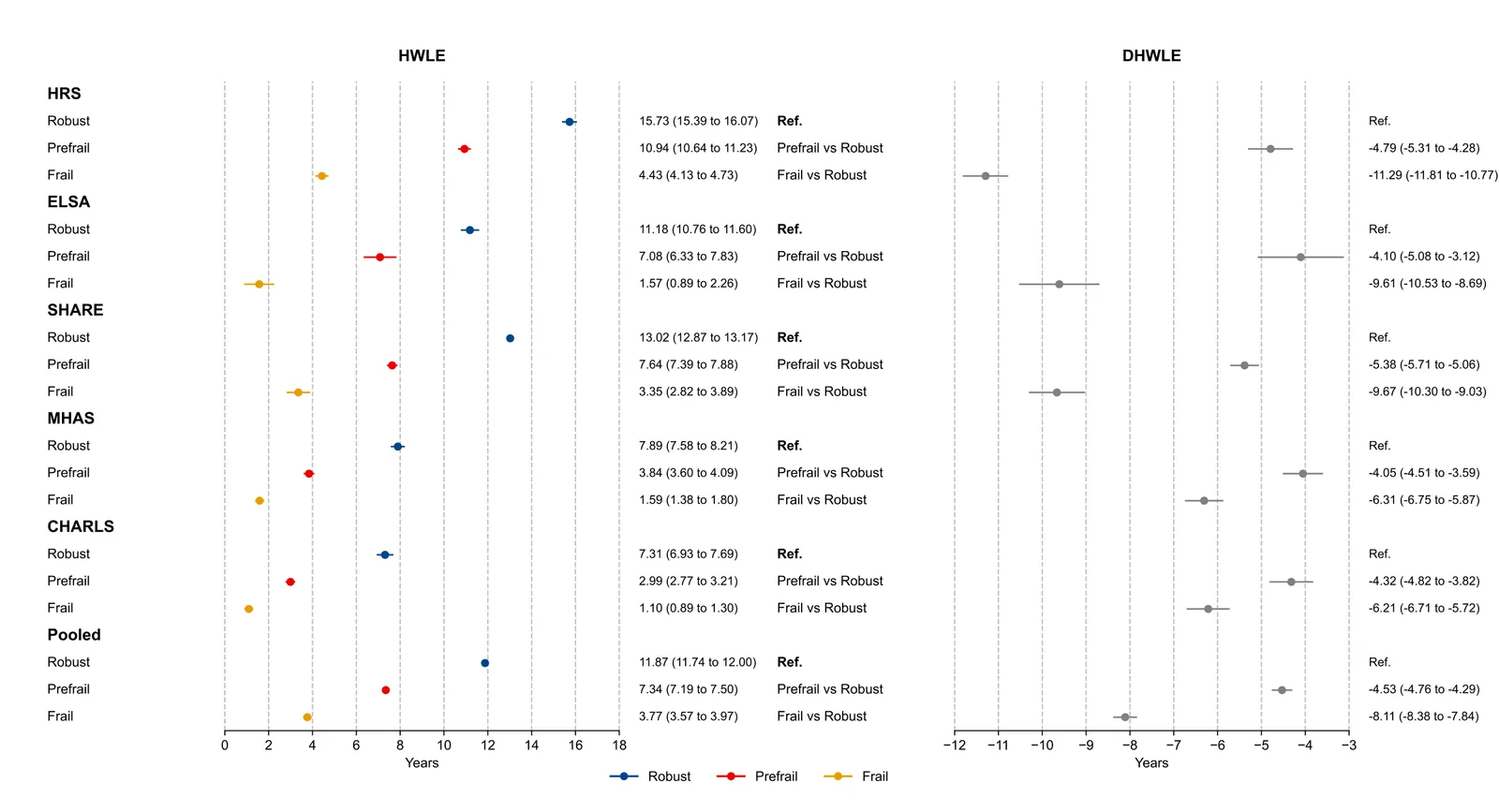
HWLE and DHWLE at age 50 years by frailty, with 95% CIs for HWLE and Bonferroni-adjusted 95% CIs for DHWLE in parentheses. CHARLS – the China Health and Retirement Longitudinal Study, CI – confidence interval, DHWLE – differences in healthy working life expectancy, ELSA – the English Longitudinal Study of Ageing, HRS – the Health and Retirement Study, HWLE – healthy working life expectancy, MHAS – the Mexican Health and Aging Study, SHARE – the Survey of Health, Ageing and Retirement in Europe.

Years of HWLE varied by cohort among the robust (7.31 to 15.73), pre-frail (2.99 to 10.94), and frail groups (1.10 to 4.43). We observed a consistent frailty gradient in HWLE across the HRS, ELSA, SHARE, MHAS, and CHARLS cohorts, with the robust group having the longest HWLE and the frail group the shortest.

### HWLE by frailty and individual-level factors

Men had longer HWLE than women among the robust (DHWLE = 0.92; 95% CI_BA_ = 0.61–1.23) and pre-fail groups (DHWLE = 1.07; 95% CI_BA_ = 0.69–1.45). Current non-smoking and higher educational attainment were associated with longer HWLE across all frailty statuses. Among the pre-frail group, unmarried individuals had longer HWLE than married individuals (DHWLE = 0.47; 95% CI_BA_ = 0.01–0.92), but there were no significant differences in other groups (**Figure 2**).

**Figure 2:**
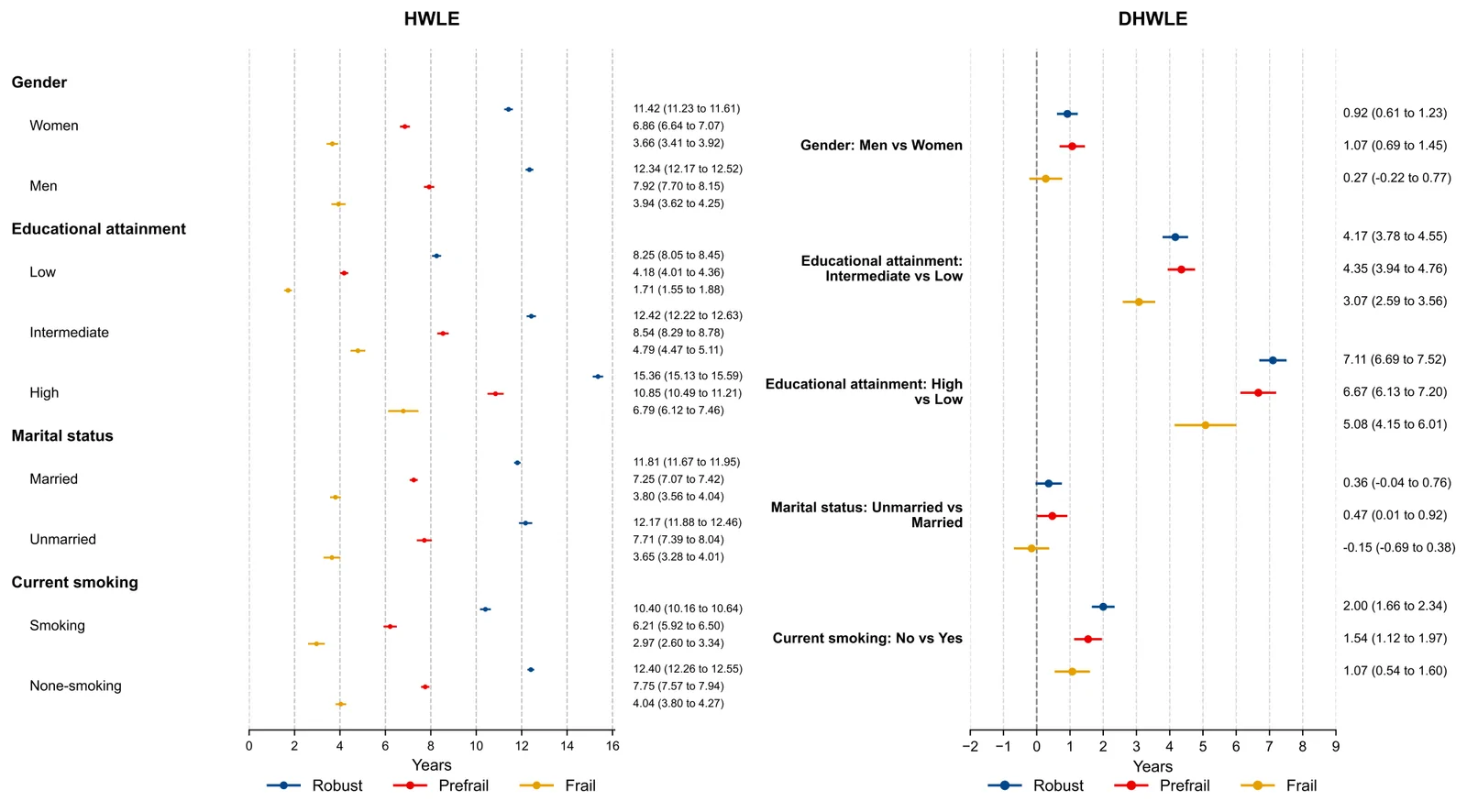
HWLE and DHWLE at age 50 years by frailty and individual-level indicators, with 95% CIs for HWLE and Bonferroni-adjusted 95% CIs for DHWLE in parentheses. CI – confidence interval, DHWLE – differences in healthy working life expectancy, HWLE – healthy working life expectancy.

### HWLE by frailty and country-level factors

Across frailty groups, participants living in countries with higher GDP *per capita*, current health expenditure, CSPI, government effectiveness, and control of corruption had longer HWLE (**Figure 3**). For example, higher GDP *per capita* was associated with 4.25 (95% CI_BA_ = 3.95–4.56), 4.57 (95% CI_BA_ = 4.21–4.93), and 2.71 (95% CI_BA_ = 2.27–3.15) additional years of HWLE in the robust, pre-frail, and frail groups. A lower Gini index and higher PSAVT were associated with longer HWLE in the robust and pre-frail groups, while a higher tax revenue was associated with longer HWLE only in the robust group.

**Figure 3:**
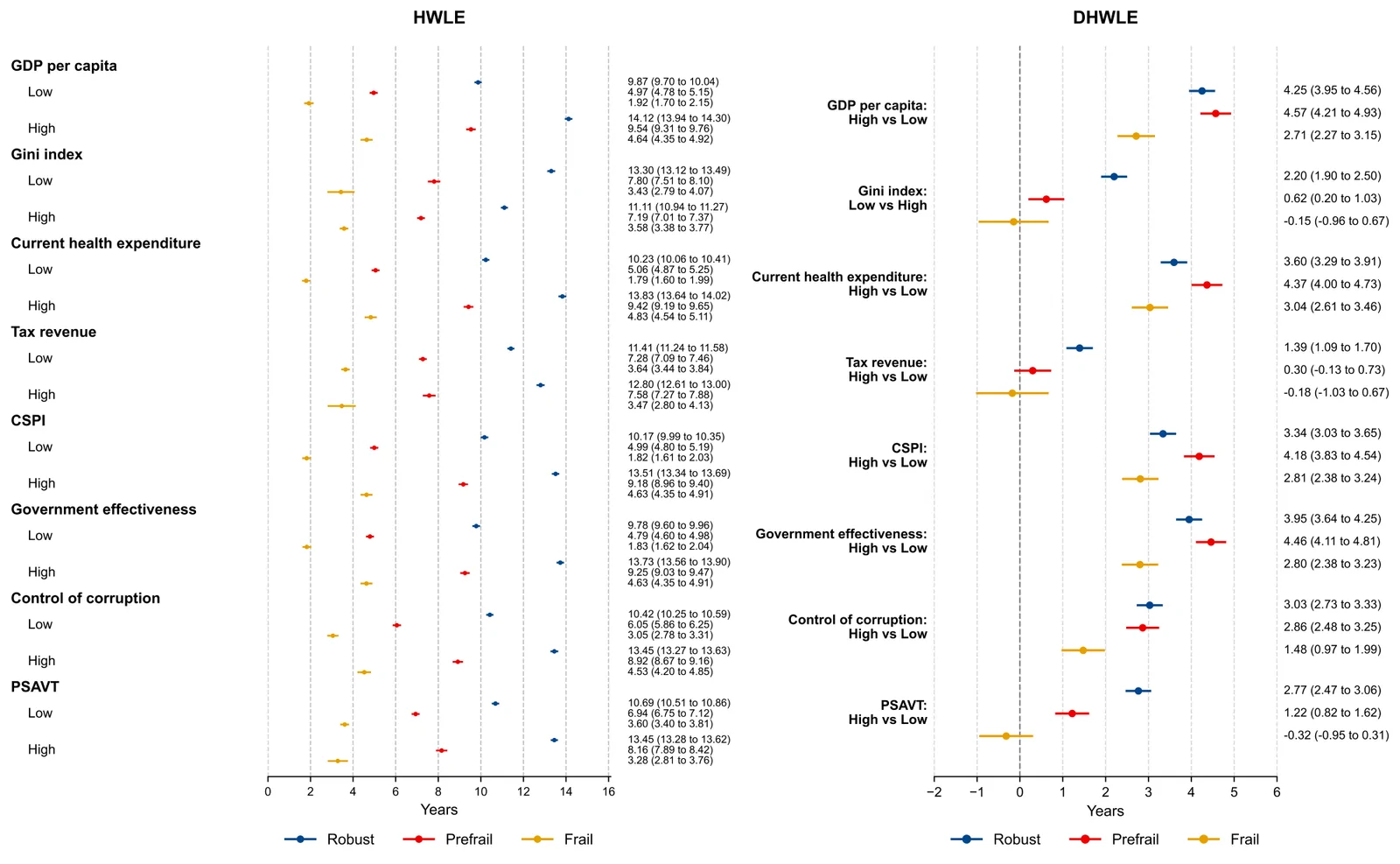
HWLE and DHWLE at age 50 years by frailty and country-level indicators, with 95% CIs for HWLE and Bonferroni-adjusted 95% CIs for DHWLE in parentheses. CI – confidence interval, CSPI – civil society participation index, DHWLE – differences in healthy working life expectancy, GDP – gross domestic product, HWLE – healthy working life expectancy, PSAVT – political stability and absence of violence/terrorism.

For the robust, pre-frail, and frail groups, participants in countries with all favourable factors had 6.65 (95% CI_BA_ = 6.11–7.19), 5.18 (95% CI_BA_ = 4.38–5.99), and 2.10 (95% CI_BA_ = 0.44–3.76) additional years of HWLE, respectively, compared with participants in countries without favourable economic and political factors. Corresponding gains associated with all favourable economic factors were 5.96 (95% CI_BA_ = 5.42–6.50), 4.64 (95% CI_BA_ = 3.85–5.43), and 1.91 (95% CI_BA_ = 0.36–3.47) years, and with all favourable political factors were 5.12 (95% CI_BA_ = 4.70–5.54), 4.53 (95% CI_BA_ = 3.98–5.08), and 1.67 (95% CI_BA_ = 0.85–2.50) years, respectively (Figure S6 in the **Online Supplementary Document**).

### Sensitivity analyses

Our results remained consistent across different interpolation steps, completeness of data, random resampling, alternative frailty cutoffs, and different methods for imputing country-level missing values, as were the estimates of the association between frailty and HWLE stratified by baseline work state, as well as the HWLE estimates across baseline frailty statuses (Table S10, Figures S7–12 in the **Online Supplementary Document**).

## DISCUSSION

To our knowledge, this is the first multicohort study to examine the association between frailty and HWLE in adults aged ≥50 years across 33 countries. We found that frailty was associated with shorter HWLE. Compared with participants in the robust group, HWLE was 4.53 years shorter in the pre-frail and 8.11 years shorter in the frail groups. Furthermore, HWLE also varied within frailty strata by individual and country-level factors.

Our HWLE estimates are broadly consistent with those put forth by previous studies. For example, HWLE at the age of 50 years was previously estimated to be 4.2 years in China [16] and approximately 9.6 years in England [13]. Our cohort-specific estimates are consistent with these findings. Previous estimates for the USA were relatively stable across cohorts, ranging from 8.6 to 8.8 years [17], but were lower than our estimates. This discrepancy may be partly explained by differences in outcome definitions, as the aforementioned study from the USA defined HWLE as the average number of years expected to be spent healthy and working between ages 51 and 80 [17], resulting in more conservative estimates. Collectively, these three studies have shown that HWLE varies across sociodemographic groups and national contexts, underscoring how it is jointly shaped by health status and labour-market participation [13,16,17]. Our findings extend this evidence by demonstrating a consistent frailty gradient in HWLE across multiple harmonised ageing cohorts.

Previous literature has demonstrated associations of frailty with adverse health outcomes and with work status as separate outcomes. We used SRH to define the health component of HWLE which, therefore, represents a key measure underlying our estimation of healthy working life. Of note, SRH reflects an individual’s physical, psychological, and social functioning and serves as a health metric that integrates objective health status with subjective psychological expectations [38]. Numerous studies have shown that frailty is a valid predictor of SRH in older adults [39–42]. Potential explanations include elevated risks of incident adverse events (*e.g.* falls and fractures) and increased mortality driven by the physical vulnerability and susceptibility associated with frailty [43], as well as reduced positive adaptive responses to external stressors [42].

Yet frailty has been shown to be substantial associated with declining labour force participation. For example, a longitudinal study utilising country-level data from 180 nations found that, after controlling for country, gender, age, and cohort fixed effects, a 1% increase in the FI was associated with a 0.6 percentage point decrease in labour force participation [44]. Multinational longitudinal research from Europe similarly indicated that poor physical health and cognitive functioning reduce employment participation among individuals aged 50 to 54 years [45]. Another study from the UK arrived at similar results [46]. Given that frailty may contribute to physical decline, poor mental health, and functional limitations, it may also be associated with precarious occupational environments for older workers. For example, a cross-sectional survey of older workers in Japan found frailty to be associated with an increased prevalence of work-related accidents, including falls, and less participation in preventive actions [47]. Longitudinal studies of European populations also observed that poor psychosocial working conditions, characterised by high effort and low reward, predicted a greater increase in frailty [48]. Similar associations have been identified among older adults in Taiwan, China [49], suggesting that positive psychosocial work conditions are beneficial for enhancing workforce health and well-being. Based on our findings and the evidence above, policies for frailty prevention and intervention should be developed alongside improvements to work environments.

Substantial individual-level inequalities in HWLE were evident across frailty statuses in our sample. Consistent with prior research [13–17], men in the robust and pre-frail groups had longer HWLE, as did individuals with higher educational attainment and current non-smokers. The gender disparities may stem from structural disadvantages women face in the labour market, including higher risks of unemployment [50], earlier statutory retirement ages in certain countries, and higher morbidity despite longer lifespans [51]. Prior evidence has indicated that smoking increases the risks of chronic conditions and mortality, highlighting the potential of smoking cessation policies and healthy lifestyle promotion in extending productive and healthy life years [52–54]. The association of education with HWLE can be interpreted through the accumulation of human capital investment and socioeconomic resources. From the life course epidemiology perspective, policy interventions should prioritise educational opportunities during childhood, a sensitive period, while encouraging adult and vocational education. Notably, our findings indicate that unmarried individuals tend to have higher HWLE than married individuals in the pre-frail group. The relationship between marital status and health remains debated, with varying disease risk profiles observed between both groups [55]. Our results in this regard align with those of several previous studies [56,57]. One possible explanation is rooted in the inclination of dyadic withdrawal, wherein social networks may contract following marriage. Conversely, unmarried individuals may maintain more diverse social ties, constituting a foundation for both health resilience and employment performance. Future studies should further validate the association between marriage and HWLE by accounting for marital duration and satisfaction, as well as by using more granular classifications.

In our analyses, favourable national economic and political factors were associated with longer HWLE across frailty statuses. These findings, however, should be interpreted as ecological and contextual associations only, as country-level indicators are likely to be highly correlated and may capture overlapping dimensions of broader socioeconomic, policy, health system, and governance environments, including the unequal distribution of power, income, products, and services [58].

Higher GDP *per capita*, lower income inequality, greater current health expenditure, higher tax revenue, stronger civil society participation, and better governance performance were generally associated with a longer HWLE, although the patterns varied across frailty statuses. These associations may reflect the contextual conditions in which older adults live, age, receive healthcare, and participate in work. Stronger economic resources and lower income inequality may reflect more favourable material and social conditions, while their absence may be associated with barriers to healthcare, education, and employment opportunities, which in turn may relate to poorer health and labour market participation. Increased health expenditure may be related to better access to healthcare services [59], and tax revenue may indicate fiscal capacity for public services, including health-promoting policies such as taxes on harmful products [60]. Higher civil society participation may reflect institutional stability and reduced income inequality, and social cohesion as collective social capital [61,62]. Better governance performance may be linked to more equitable and efficient health systems [63], and to lower unemployment associated with trust in government effectiveness and policy efficiency [64].

Robust and pre-frail individuals derived greater benefits in HWLE from these favourable country-level factors, which highlights the importance of timely prevention and intervention of frailty progression. Economic and political development policies should be integrated with healthy ageing strategies focused on frailty prevention and intervention, thereby extending HWLE by facilitating positive transitions in frailty statuses.

Our findings have important policy implications. Given that frailty can affect older adults across populations, health systems should integrate frailty screening into primary and acute care, develop integrated care tailored to local socioeconomic contexts, and prioritise financing for frailty identification and intervention programmes. Our results also point to a need for multisectoral action in response to population ageing. Policymakers should prioritise women’s health and work-related rights, strengthen tobacco-control policies and their implementation, and invest in governance and social protection systems. Employers should provide age-friendly workplaces for older workers, including flexible schedules and work arrangements, supportive workplace networks that foster social capital, and occupational health protections. Such coordinated efforts may help delay frailty progression while promoting healthier, voluntary, and supported work participation in later life.

The key strength of this study is our use of five large cohorts to examine the association between frailty and HWLE. We also accounted for the temporal dynamics of both the core exposure and the outcome simultaneously, which mitigated the misclassification bias that arises when relying solely on the baseline characteristic for frailty. Furthermore, we explored both individual and national-level inequalities in healthy working life expectancy across various frailty statuses.

However, several limitations should be noted. First, the presence of cohort effects may have introduced potential confounding. Considering the model complexity constraints, a birth cohort was not included in our analytical models. Second, although variables were harmonised across cohorts as far as possible, residual measurement non-equivalence cannot be fully excluded. Despite standard FI construction, cohort differences in deficit items may have introduced misclassification bias. Health was measured using SRH, which may be influenced by cross-national sociocultural differences. Work was defined as encompassing paid work and self-employment, which may not fully capture economic contribution. Accordingly, future studies using more detailed and comparable measures are needed to further validate HWLE estimates across diverse settings. Third, for individuals recorded as deceased during a given wave, but with an unknown exact date of death, we imputed the date using the midpoint between their last two waves. Although this approach is widely adopted in epidemiological studies [16,26,28], it still may have introduced information bias. Fourth, although our findings are based on 33 countries across 4 continents, the absence of cohorts from Africa, South Asia, the Middle East, and Oceania may limit the extrapolation of the results to populations in these regions. Future HWLE-related research should prioritise low-income and underrepresented settings, where labour market structures, healthcare access, retirement systems, and social protection may differ substantially. Fifth, the healthy worker effect may have biased the results, as community-dwelling older adults included in this study were likely healthier than institutionalised ones and more likely to remain in the labour market, potentially leading to overestimation of HWLE. Finally, we did not quantify the marginal effects of improvements in country-level factors on HWLE across frailty groups.

## CONCLUSIONS

Based on a multicohort design and a multinational perspective, we found that frailty was associated with shorter HWLE. Frail 50-year-olds had more than eight fewer years of HWLE than their peers in the robust group, representing a substantial loss of human capital and economic productivity. Individual and country-level inequalities were evident in HWLE across frailty statuses. These findings highlight the importance of integrating frailty prevention and intervention programmes with broader initiatives to promote economic, political, and population development. Given the reversible nature of frailty, effective prevention and intervention programmes have the potential to substantially extend healthy working life among older adults worldwide. Achieving this goal will require concerted efforts across multiple sectors of society and close collaboration within the international community at large to better support older adults in working and living longer, healthier lives.

## Additional material


Online Supplementary Document

## Data Availability

**Data availability:** The analysis uses data and information from the RAND HRS dataset and codebook, version 1 (as of May 2025); the Harmonized HRS dataset and codebook, version D (as of August 2023); the Harmonized ELSA dataset and codebook, version G.2 (as of July 2021); the Harmonized SHARE dataset and codebook, version G (as of October 2024); the Harmonized MHAS dataset and codebook, Version D (as of May 2025); and the Harmonized CHARLS dataset and codebook, version D (as of June 2021). All datasets and codebooks were developed by the Gateway to Global Aging Data. The development of the Harmonized datasets was funded by the National Institute on Aging (R01 AG030153, RC2 AG036619, R03 AG043052, U01 AG009740, R01 AG073289) and the Social Security Administration. For more information, please refer to the following link: https://g2aging.org/. Data on country-level indicators can be found at: https://data.worldbank.org/; https://v-dem.net/about/v-dem-project/.
