# Supplementary material for: Frailty and healthy working life expectancy: a multicohort modelling study
Source: J Glob Health. 2026 Oct 19;16:04338. doi: 10.7189/jogh.16.04338 (PMC13679354; doi:10.7189/jogh.16.04338)
Supplement: Supplementary file 1 [file jogh-16-04338-s001.pdf]

Supplement to: Shui Y, Zhu J, Huang Z, You H, Gao J. Frailty and healthy working life expectancy: a multicohort modelling study. J Glob Health. 2026;16:04338

Contents

Appendix S1. Details on the construction of frailty index across cohorts
Appendix S2. Definitions of individual and country-level factors
Appendix S3. Details on the model selection under the hypothesis of discrete-time Markov chains
Appendix S4. Inverse probability weighting sensitivity analysis
Table S1. STROBE reporting guidelines items
Table S2. Adherence to JoGH's GRABDROP guidelines items
Table S3. Comparison of survey wording for self-rated health
Table S4. Comparison of construction for work state
Table S5. Indicators on the construction of frailty index across cohorts
Table S6. Distribution of frailty index across cohorts at baseline
Table S7. Interaction results based on likelihood ratio tests
Table S8. Basic characteristics of included and excluded participants
Table S9. Health working related life expectancy at age 50 years by frailty
Table S10. Imputed values for missing information on country-level factors through differential imputed methods
Figure S1. Transitions among five healthy working related states
Figure S2. Estimated annual transition probabilities among five healthy working related states by frailty status at age 50 years
Figure S3. Distribution of frailty index across cohorts at baseline
Figure S4. Process of sample selection
Figure S5. HWLE at age 50 years across cohorts
Figure S6. HWLE and DHWLE at age 50 years by frailty and numbers of favourable country-level indicators
Figure S7. Comparison of HWLE by frailty through the differential methods
Figure S8. Comparison of HWLE by frailty under the differential cutoffs of frailty status
Figure S9. Comparison of the imputed values through alternative imputation methods
Figure S10. Number of country-year dichotomous information inconsistent with imputation results in the main analysis (M1)

Figure S11. Comparison of HWLE by frailty through alternative imputation methods
Figure S12. HWLE by baseline frailty status and stratified by baseline work state

Appendix S1. Details on the construction of frailty index across cohorts

Step 1. Selection of health deficits

The selected health deficits possessed the following characteristics [1]:

(1) they covered a range of organ systems or domains of functioning;

(2) their prevalence generally increased with age;

(3) they must not saturate too early; and

(4) their prevalence should not be lower than 1%.

The minimum number of health deficits included was 30. Each deficit variable was transformed into a single score ranging from 0 (no deficit) to 1 (most severe). Items of selected health deficits across cohorts can be found in **Table S5**.

Step 2. Calculation for the frailty index (FI)

The sum of health deficits present was then divided by the total number of deficit items selected, yielding a continuous variable ranging from 0 to 1 with higher scores indicating higher degree of frailty, known as the score of FI.

Step 3. Categorization for the FI

The FI was further categorized into three levels of frailty according to the previous literature [2]:

(1) Robust (FI score ≤ 0.10),

(2) Prefrail (FI score > 0.10 to < 0.25), and

(3) Frail (FI score ≥ 0.25).

References

1. Hoogendijk EO, Afilalo J, Ensrud KE, Kowal P, Onder G, Fried LP. Frailty: implications for clinical practice and public health. Lancet. 2019;394:1365-75.

2. Fan J, Yu C, Guo Y, Bian Z, Sun Z, Yang L, et al. Frailty index and all-cause and cause-specific mortality in Chinese adults: a prospective cohort study. Lancet Public Health. 2020;5:e650-e60.

Appendix S2. Definitions of individual and country-level factors

Individual-level factors

(1) Gender: *Men* or *women* based on self-reported by participants.

(2) Educational attainment: Educational attainment is categorized according to a three-tier harmonized scale which is a simplified version of 1997 International Standard Classification of Education (ISCED-97) codes, with levels coded as follows: *less than upper secondary education (low)*, *upper secondary & vocational training (intermediate)*, and *tertiary education (high)*.

(3) Marital status: Marital status is created using current marital status, with states dichotomized into *married* (including married and partnered) and *unmarried* (including separated, divorced, widowed, and never married).

(4) Current smoking: Current smoking behaviour is dichotomized into *currently smoking (Yes)* and *currently not smoking (No)* based on participants' response to the question whether currently smoke cigarettes.

Country-level factors

Economic domain

(1) GDP per capita (constant 2015 US$): GDP per capita is gross domestic product divided by the general population, which is expressed in constant 2015 U.S. dollars.

(2) Gini index: Gini index measures the extent to which the distribution of income (or, in some cases, consumption expenditure) among individuals or households within an economy deviates from a perfectly equal distribution. A Lorenz curve plots the cumulative percentages of total income received against the cumulative number of recipients, starting with the poorest individual or household. The Gini index measures the area between the Lorenz curve and a hypothetical line of absolute equality, expressed as a percentage of the maximum area under the line. A Gini index of 0 represents perfect equality, while an index of 100 implies perfect inequality.

(3) Current health expenditure (% of GDP): Level of current health expenditure expressed as a percentage of GDP. Estimates of current health expenditures include healthcare goods and services consumed during each year. This indicator does not include capital health expenditures such as buildings, machinery, IT and stocks of vaccines for emergency or outbreaks.

(4) Tax revenue (% of GDP): Taxes are compulsory, unrequited payments, in cash or in kind, made by institutional units to government units. Tax revenue is expressed as a percentage of GDP.

Political domain

(5) Civil society participation index (CSPI): Based on the expert estimates and index by The Varieties of Democracy (V-Dem). CSPI is designed to provide a measure of a robust civil society, understood as one that enjoys autonomy from the state and in which citizens freely and actively pursue their political and civic goals, however conceived. It ranges from 0 (low) to 1 (high).

(6) Government effectiveness: Government effectiveness captures perceptions and views of the quality of public services, the quality of the civil service and the degree of its independence from political pressures, the quality of policy formulation and implementation, and the

credibility of the government's commitment to such policies. It ranges from -2.5 (low) to 2.5 (high).
(7) Control of corruption: Control of Corruption captures perceptions of the extent to which public power is exercised for private gain, including both petty and grand forms of corruption, as well as "capture" of the state by elites and private interests. It ranges from -2.5 (low) to 2.5 (high).
(8) Political stability and absence of violence/terrorism (PSAVT): PSAVT measures perceptions of the likelihood of political instability and/or politically-motivated violence, including terrorism. It ranges from -2.5 (low) to 2.5 (high).

Individual-level factors were collected at baseline, while country-level factors were collected in the preceding year of baseline.

Appendix S3. Details on the model selection under the hypothesis of discrete-time Markov chains

Concrete information on calculation of transition probabilities among health-related states and healthy expectancies under the discrete-time multistate Markov model has been described in the previous publication [1], which was summarized and applied as follows in this study.

We defined five health and work related states, i.e. healthy and not working (State 1: HnW), healthy and working (State 2: HW), not healthy and not working (State 3: nHnW), not healthy and working (State 4: nHW), or death (State 5), with the first four states entitled to transform into one another and death serving as the absorbing state (**Figure S1**).

Estimating transition probabilities

Let $X(x)$ denote the state of an individual aged x. After time h this individual is in state $X(x+h)$. Assume that $X(x)$ is a non-homogeneous discrete parameter Markov chain on these five states with transition probabilities:

$$ {}_hp_x^{jk} = \Pr(X(x+h) = k | X(x) = j) \tag{1}$$

and one step transition matrix:

$$ {}_hP_x = ({}_hp_x^{jk}) = \begin{pmatrix} {}_hp_x^{11} & {}_hp_x^{12} & {}_hp_x^{13} & {}_hp_x^{14} & {}_hp_x^{15} \\ {}_hp_x^{21} & {}_hp_x^{22} & {}_hp_x^{23} & {}_hp_x^{24} & {}_hp_x^{25} \\ {}_hp_x^{31} & {}_hp_x^{32} & {}_hp_x^{33} & {}_hp_x^{34} & {}_hp_x^{35} \\ {}_hp_x^{41} & {}_hp_x^{42} & {}_hp_x^{43} & {}_hp_x^{44} & {}_hp_x^{45} \\ 0 & 0 & 0 & 0 & 1 \end{pmatrix} \tag{2}$$

A natural parametrization for the transition probabilities is the multinomial logit. The time unit h is set to biennium and the following analysis assumes this premise by default. The simple model is:

$$ \ln \frac{{}_hp_x^{jk}}{{}_hp_x^{jj}} = \alpha_{jk}(h) + \beta_{jk}(h)x, \qquad j \neq k \tag{3}$$

where x denotes individual age, α_{jk} and β_{jk} are parameters to be estimated.

Identified parameters to be estimated

$$ \ln \frac{{}_hP_x^{jk}}{{}_hP_x^{jj}} = \alpha_{jk}(h) + \beta_{jk}(h)AGE + \gamma_{jk}(h)PREFRAIL + \delta_{jk}(h)FRAIL, \qquad j \neq k \tag{4}$$

Equation 4 (E4) denotes the simplest form of the model in this study. We applied this equation within each stratum of each subpopulation identifier to estimate the effect of frailty on transitions in health and work states. $\alpha_{jk}(h)$ is the intercept, $\beta_{jk}(h)$ estimates the effect of *age* on state transitions. $\gamma_{jk}(h)$ and $\delta_{jk}(h)$ measures the estimated effect of *Prefrail* and *Frail* status compared to the *Robust* status respectively.

$$\ln \frac{{}_hP_x^{jk}}{{}_hP_x^{jj}} = \alpha_{jk}(h) + \beta_{jk}(h)AGE + \gamma_{jk}(h)PREFRAIL + \delta_{jk}(h)FRAIL + \varepsilon_{jk}(h)COV \tag{5}$$

Equation 5 (E5) further adds a covariate (subpopulation identifier) based on E4. $\varepsilon_{jk}(h)$ measures the estimated effect of *the specific dummy covariate.* The dummy covariate can be the individual or country level, which is merely allowed to include two classes. For polytomus covariates with n classes, they are designed to create $n-1$ dummy variables.

$$\ln \frac{{}_hP_x^{jk}}{{}_hP_x^{jj}} = \alpha_{jk}(h) + \beta_{jk}(h)AGE + \gamma_{jk}(h)PREFRAIL + \delta_{jk}(h)FRAIL + \varepsilon_{jk}(h)COV + \vartheta_{jk}(h)COV \times PREFRAIL + \mu_{jk}(h)COV \times FRAIL \tag{6}$$

Based on the E5, Equation 6 (E6) further adds interactions comprising the product of covariate and prefrail status, and product of covariate and frail status. We use the likelihood ratio tests (LRTs) based on E5 and E6 (nested models) to identify the statistical significance of interactions. LRT results are reported in **Table S7**.

Estimating health expectancy

If the initial health and work state is j at age $x-t$, the stable prevalences (i.e. period prevalences) of the outcome state being 1 (HnW), 2 (HW), 3 (nHnW) and 4 (nHW) at age x are:

$$_tw^{jk}(x) = \frac{_tp_{x-t}^{jk}}{\sum_{u=1}^{4} {}_tp_{x-t}^{ju}} \qquad k = 1,2,3,4 \tag{6}$$

For fixed x assume that

$$0 \le b_t = \min_{j,k} {}_tp_x^{jk} < \max_{j,k} {}_tp_x^{jk} = B_t \tag{7}$$

with at most a finite number of b_t zero. Then, uniformly in j,

$$\lim_{t\to\infty} {}_tw^{jk}(x) = w^k(x) \qquad k = 1,2,3,4 \tag{8}$$

exists, $\sum_k w^k(x) = 1$, provided $\sum_{t=2}^{\infty}(b_t b_{t-1}/B_t B_{t-1})$ diverges. w_x^k is a fraction of people in state k at age x.

For individuals at age x:

$$\sum_{k=1}^{4} w^k(x) = 1 \tag{9}$$

$$w^2(x) = 1 - (w^1(x) + w^3(x) + w^4(x)) \tag{10}$$

where $w^2(x)$ denotes the fraction of people with the state of healthy and working at age x. The transition probability to state k at age x regardless of the initial status denotes:

$$_yp_x^k(\theta) = \sum_{j=1}^{4} w^j(x,\theta)\ {}_yp_x^{jk}(\theta) \tag{11}$$

where $w^j(x,\theta) = w^j(x)$ with θ denoting the vector of parameters.

The health working related expectancy of state k from the initial state j at age x is:

$$e_x^{jk} = \sum_{y=1}^{\infty} {}_y p_x^{jk} \tag{12}$$

The health working related expectancy from the initial state j at age x over the interval $(x, x + y)$ is:

$${}_y e_x^{jk} = \sum_{u=1}^{y} {}_u p_x^{jk} \tag{13}$$

where y denotes the upper limit in the sums.
The health working related expectancy of state k at age x is:

$$e_x^{\cdot k}(\theta) = \sum_{j=1}^{4} w^j(x,\theta) e_x^{jk}(\theta) = \sum_{y=0}^{\infty} {}_y p_x^{\cdot k}\left(\theta, w^2(x,\theta)\right) \tag{14}$$

The total life expectancy at age x is the sum of health working related expectancies of state k:

$$e_x^{\cdot\cdot}(\theta) = \sum_{k=1}^{4} e_x^{\cdot k}(\theta) \tag{15}$$

Estimation for transition probabilities and health expectancy which are mentioned above is based on maximum likelihood estimation.

Reference

1. Lièvre A, Brouard N, Heathcote C. The estimation of health expectancies from cross-longitudinal surveys. Math Popul Stud. 2003;10:211-48.

Appendix S4. Inverse probability weighting sensitivity analysis

We conducted an individual-level inverse probability weighting (IPW) sensitivity analysis to assess whether person-level selection into the analytic sample influenced the estimates [1]. The primary analyses were unweighted. Because the multistate modelling software (IMaCh) allowed only one weight per participant, the IPW analysis addressed selection into the analytic sample rather than interval-specific attrition or time-varying missingness.

Three sequential retention indicators were defined according to the sample selection process: $R_1 = 1$ indicated that the participant was not excluded because of missing frailty records across all alive waves; $R_2 = 1$ indicated that the participant was not excluded because of missing or single-wave records for health and work status; and $R_3 = 1$ indicated that the participant was not excluded because of missing subpopulation identifiers. Indicators were coded as 1 for retained and 0 for excluded. Participants with implausible death-date records were rare and were treated as data-quality exclusions rather than as a separate weighting step.

For each exclusion step, we estimated stabilized inverse probability selection weights [1, 2]. The denominator model estimated the probability of being retained conditional on baseline age, gender, and cohort, whereas the numerator model included cohort only. Thus, the step-specific weights were defined as:

$$w_{R_1} = \frac{P(R_1 = 1 \mid cohort)}{P(R_1 = 1 \mid gender, age, cohort)}$$

$$w_{R_2} = \frac{P(R_2 = 1 \mid R_1 = 1, cohort)}{P(R_2 = 1 \mid R_1 = 1, gender, age, cohort)}$$

$$w_{R_3} = \frac{P(R_3 = 1 \mid R_1 = 1, R_2 = 1, cohort)}{P(R_3 = 1 \mid R_1 = 1, R_2 = 1, gender, age, cohort)}$$

The final stabilized selection weight was calculated as:

$$w_{selection} = w_{R_1} \times w_{R_2} \times w_{R_3}$$

Weight truncation was used to reduce the influence of extreme weights. Extreme weights were truncated at the 1st and 99th percentiles and then standardized to have a mean of 1 before being incorporated into the multistate Markov models.

References

1. Seaman SR, White IR. Review of inverse probability weighting for dealing with missing data. Stat Methods Med Res. 2013;22:278-95.

2. Austin PC, Stuart EA. Moving towards best practice when using inverse probability of treatment weighting (IPTW) using the propensity score to estimate causal treatment effects in observational studies. Stat Med. 2015;34:3661-79.

Table S1. STROBE reporting guidelines items

	Item No	Recommendation	Page No
**Title and abstract**	1	(*a*) Indicate the study's design with a commonly used term in the title or the abstract<br>(*b*) Provide in the abstract an informative and balanced summary of what was done and what was found	(*a*) p 1 Abstract<br>(*b*) p 1 Abstract
**Introduction**			
Background/rationale	2	Explain the scientific background and rationale for the investigation being reported	pp 2-3 Introduction
Objectives	3	State specific objectives, including any prespecified hypotheses	p 1 Abstract<br>p 3 Introduction
**Methods**			
Study design	4	Present key elements of study design early in the paper	pp 3-5 Methods
Setting	5	Describe the setting, locations, and relevant dates, including periods of recruitment, exposure, follow-up, and data collection	p 3 Methods<br>Figure S1
Participants	6	(*a*) Give the eligibility criteria, and the sources and methods of selection of participants. Describe methods of follow-up<br>(*b*) For matched studies, give matching criteria and number of exposed and unexposed	*(a)* p 3 Methods<br>Figure S1<br>*(b)* NA
Variables	7	Clearly define all outcomes, exposures, predictors, potential confounders, and effect modifiers. Give diagnostic criteria, if applicable	pp 4-5 Methods
Data sources/ measurement	8*	For each variable of interest, give sources of data and details of methods of assessment (measurement). Describe comparability of assessment methods if there is more than one group	pp 4-5 Methods<br>Data availability<br>Appendices S1-2 and Tables S4-7
Bias	9	Describe any efforts to address potential sources of bias	pp 4-6 Methods<br>p 13 Discussion<br>Appendix S4
Study size	10	Explain how the study size was arrived at	NA
Quantitative variables	11	Explain how quantitative variables were handled in the analyses. If applicable, describe which groupings were chosen and why	pp 4-6 Methods<br>Appendices S1-2
Statistical methods	12	(*a*) Describe all statistical methods, including those used to control for confounding<br>(*b*) Describe any methods used to examine subgroups and interactions	*(a)* pp 5-6 Methods<br>Appendix S3<br>*(b)* pp 5-6 Methods<br>Appendix S3

	Item No	Recommendation	Page No
		(*c*) Explain how missing data were addressed	*(c)* pp 4-6 Methods<br>Appendix S4
		(*d*) If applicable, explain how loss to follow-up was addressed	*(d)* Appendix S4
		(*e*) Describe any sensitivity analyses	*(e)* p 6 Methods<br>p 9 Results<br>Appendix S4<br>Table S10 Figures S7-12
**Results**			
Participants	13*	(a) Report numbers of individuals at each stage of study—eg numbers potentially eligible, examined for eligibility, confirmed eligible, included in the study, completing follow-up, and analysed	*(a)* p 7 Results<br>Figure S1
		(b) Give reasons for non-participation at each stage	*(b)* Figure S1
		(c) Consider use of a flow diagram	*(c)* Figure S1
Descriptive data	14*	(a) Give characteristics of study participants (eg demographic, clinical, social) and information on exposures and potential confounders	*(a)* p 7 Results<br>Table 1
		(b) Indicate number of participants with missing data for each variable of interest	*(b)* Figure S1<br>Table S3
		(c) Summarise follow-up time (eg, average and total amount)	*(c)* p 7 Results
Outcome data	15*	Report numbers of outcome events or summary measures over time	NA
Main results	16	(*a*) Give unadjusted estimates and, if applicable, confounder-adjusted estimates and their precision (eg, 95% confidence interval). Make clear which confounders were adjusted for and why they were included	*(a)* p 1 Abstract<br>pp 7-8 Results<br>Figures 1-3<br>Table S9 and Figure S6
		(*b*) Report category boundaries when continuous variables were categorized	*(b)* pp 4-5 Methods
		(*c*) If relevant, consider translating estimates of relative risk into absolute risk for a meaningful time period	*(c)* NA
Other analyses	17	Report other analyses done—eg analyses of subgroups and interactions, and sensitivity analyses	pp 8-9 Results<br>Figures 2-3<br>Table S10 and Figures S7-12
**Discussion**			
Key results	18	Summarise key results with reference to study objectives	p 9 Discussion

	Item No	Recommendation	Page No
			p 14 Conclusions
Limitations	19	Discuss limitations of the study, taking into account sources of potential bias or imprecision. Discuss both direction and magnitude of any potential bias	pp 13-14 Discussion
Interpretation	20	Give a cautious overall interpretation of results considering objectives, limitations, multiplicity of analyses, results from similar studies, and other relevant evidence	pp 9-13 Discussion
Generalisability	21	Discuss the generalisability (external validity) of the study results	p 14 Discussion
**Other information**			
Funding	22	Give the source of funding and the role of the funders for the present study and, if applicable, for the original study on which the present article is based	Funding

Table S2. Adherence to JoGH's GRABDROP guidelines items

JoGH guideline items
1. Please list all papers published by each co-author in previous 3 years that were based on secondary analysis of a big data repository
Zhu J, Huang Z, Yang S, Jiang J, You H, Gao J. Transitions of loneliness and lonely life expectancy: A longitudinal study across 24 countries. Int Psychogeriatr. 2025:100134.<br>Gao J, Chen H, Chen H, Chen Y, Xu J, Wang Y, et al. A cohort study of self-perception of ageing and all-cause mortality among older adults in China: a multiple mediators analysis. BMC Public Health. 2024;24:1382.<br>Ye B, Wang Y, Xu J, Jiang J, Yang S, Chen J, et al. How long were older people expected to live with or without sarcopenia? Multistate modeling of a national cohort study. Front Public Health. 2023;11:1203203.<br>Gao J, Wang Y, Xu J, Jiang J, Yang S, Xiao Q. Life expectancy among older adults with or without frailty in China: multistate modelling of a national longitudinal cohort study. BMC Med. 2023;21:101.
2. Please explain the key elements of your study design and the use of the available datasets that make your study an original scientific contribution
In this multicohort modelling study, we used discrete-time multistate Markov models to explore the association between frailty and healthy working life expectancy (HWLE) among more than 180000 participants aged 50 years and above from five harmonized health and retirement cohorts.<br>We simultaneously accounted for the temporal dynamics of both the core exposure and the outcome, which mitigated the misclassification bias that arises when relying solely on the baseline frailty status. Furthermore, we explored both individual and country-level inequalities in HWLE across various frailty statuses.<br>We found that frailty was associated with shorter HWLE, and individual and country-level inequalities were evident in HWLE across frailty statuses. The evidence highlights the need to integrate targeted frailty prevention and intervention programs with broader initiatives aimed at fostering national economic and political development, thereby promoting healthier, voluntary, and supported work participation in later life.
3. Please list all publications that addressed similar research questions in the same dataset and indicate where you cited them in your paper
Several previous studies estimated healthy working life expectancy in individual countries and investigated HWLE disparities by gender, birth cohort, educational attainment, lifestyle, and chronic disease. We have cited these studies in the Introduction section.<br>Parker M, Bucknall M, Jagger C, Wilkie R. Population-based estimates of healthy working life expectancy in England at age 50 years: analysis of data from the English Longitudinal Study of Ageing. Lancet Public Health. 2020;5:e395-e403.<br>Hambisa MT, Tawiah R, Jagger C, Kiely KM. Gender, education, and cohort differences in healthy working life expectancy at age 50 years in Australia: a longitudinal analysis. Lancet Public Health. 2023;8:e610-e7.<br>Sperlich S, Beller J, Epping J, Geyer S, Tetzlaff J. Trends of healthy and unhealthy working life expectancy in Germany between 2001 and 2020 at ages 50 and 60: a question of educational level? J Epidemiol Community Health. 2023;77:430-9.<br>Li C, Wang L, Ding L, Zhou Y. Determinants and inequities in healthy working life expectancy in China. Nat Med. 2024;30:3318-26.<br>Blain F, Boissonneault M. Healthy working life expectancy across birth cohorts in the United States. J Gerontol B Psychol Sci Soc Sci. 2025;80:gbaf119.<br>Wilkie R, Onamusi O, Potts J, Lynch M. How long do people with rheumatic and musculoskeletal diseases stay healthy and in work across Europe? Analysis of data from SHARE and ELSA. Ann Rheum Dis. Forthcoming 2026.

A previous study explored the association between individual and country-level factors and lonely life expectancy at age 60 based on a multicohort design. We have cited this study in the Methods section.<br>Zhu J, Huang Z, Yang S, Jiang J, You H, Gao J. Transitions of loneliness and lonely life expectancy: A longitudinal study across 24 countries. Int Psychogeriatr. 2025:100134.
4. Please explain how you addressed multiple testing through an appropriately rigorous statistical threshold and indicate this in the methods section
When comparing HWLE across groups, we applied Bonferroni correction to address the potential issue of false positives due to multiple comparisons. Bonferroni-corrected confidence intervals were provided for all differences in HWLE, as clearly stated in the Statistical analyses of the Methods.
5. Please declare to what extent have AI chatbots been used in developing your paper and to which parts of the paper did they contribute
No AI chatbots were used in the conceptualization, data acquisition, study design, statistical analyses, interpretation of results, or drafting of the manuscript. All authors reviewed and approved the final submitted text and remain fully responsible for the manuscript content.

Table S3. Comparison of survey wording for self-rated health

Cohort	Years of data used in study	Survey question	Response scales
HRS	2004, 2006, 2008, 2010, 2012, 2014, 2016, 2018, 2020, 2022	Would you say your health is excellent, very good, good, fair, or poor?	1.Excellent<br>2.Very good<br>3.Good<br>4.Fair<br>5.Poor
ELSA	2002, 2004, 2008, 2010, 2012	Would you say your health is … ?	1.Excellent<br>2.Very good<br>3.Good<br>4.Fair<br>5.Poor
SHARE	2004, 2006, 2010, 2013, 2015, 2017, 2019, 2021	Would you say your health is ... ?	1.Excellent<br>2.Very good<br>3.Good<br>4.Fair<br>5.Poor
MHAS	2001, 2003, 2012, 2015, 2018, 2021	Now I have some questions about your health.<br>Would you say your health is... ?	1.Excellent<br>2.Very good<br>3.Good<br>4.Fair<br>5.Poor
CHARLS	2011, 2013, 2015, 2018	Would you say your health is (response scale listed)?	1.Very good<br>2.Good<br>3.Fair<br>4.Poor<br>5.Very poor

Table S4. Comparison of construction for work state

Cohort	Years of data used in study	Variable construction in harmonized cohorts
HRS	2004, 2006, 2008, 2010, 2012, 2014, 2016, 2018, 2020, 2022	**Work state** is derived from the question: "Are you currently working for pay?", which recodes this variable to a yes/no indicator.
ELSA	2002, 2004, 2008, 2010, 2012	**Work state** is derived from a set of questions about activities done over the past month. A value of 0 indicates that the respondent is not currently doing paid work and a value of 1 indicates that the respondent is currently doing paid work.
SHARE	2004, 2006, 2010, 2013, 2015, 2017, 2019, 2021	In 2004 and for new respondents in subsequent waves, **work state** indicates whether the individual did any paid work in the last four weeks. Starting from 2006, **work state** indicates whether the individual did any paid work since the last interview for previously interviewed respondents.<br>**Work state** takes a value of 1 if the individual did work for pay and 0 otherwise.
MHAS	2001, 2003, 2012, 2015, 2018, 2021	**Work state** indicates whether the respondent is working in the current wave.<br>**Work state** is derived from the question in 2001: 'Last week did you... Worked, Did not work but you had a job, Looked for work, Were a student, Dedicated self to household chores, or Did not work?'. The 'Worked' and 'Did not work but had a job' answers were treated as currently 'Working for pay'. The rest of the answers were treated as 'Not working for pay'.<br>Starting from 2003, **work state** was derived from the question: 'Are you currently... Working, Looking for work, or Don't work'. The 'Looking for work' and 'Don't work' answers were treated as currently 'Not Working for pay'.<br>**Work state** is coded as 0 if the respondent is not working and as 1 if the respondent is working.
CHARLS	2011, 2013, 2015, 2018	**Work state** indicates whether the respondent engaged in any work in the past year, which is derived from the labour force status variable.<br>**Work state** is assigned 0 if the respondent is unemployed, retired, or never worked per their labour force status.<br>**Work state** is assigned 1 if the respondent reports engaging in agricultural work, non-agricultural employed work, non-agricultural self-employed work, or non-agricultural unpaid family business work per their labour force status.

Table S5. Indicators on the construction of frailty index across cohorts

Item	Whether used in this study					Threshold
	HRS	ELSA	SHARE	MHAS	CHARLS	
	Wave 7-16	Wave 1-2, 4-6	Wave 1-2, 4-9	Wave 1-6	Wave 1-4	
Depressive symptoms	√	√	√	√	√	Continuous, from 0 to 1
CES-D 8 score/8	●	●				
CES-D 9 score/9				●		
CES-D 10 score/10					●	
EURO-D 12 score/12			●			
Self-rated memory	△	△	△	△	√	Excellent=0, Very good=0.25, Good=0.5, Fair=0.75, Poor=1
Self-report of eyesight	√			√		Excellent=0, Very good=0.25, Good=0.5, Fair=0.75, Poor or Blind=1
Self-report of distance eyesight		√	√			
Self-report of near eyesight		√	√			
Self-rated hearing	√	√	√	√		Excellent=0, Very good=0.25, Good=0.5, Fair=0.75, Poor or Legally deaf =1
Underweight based on self-reported body mass index (BMI)	√	△	√	√	△	BMI $\geq$ 30 (or 25 for Asians) kg/m^2 =1
Obesity based on self-reported BMI	√	△	√	√	△	BMI < 18.5 kg/m^2 =1
Ever had high blood pressure	√	√	√	√	√	Yes=1, No=0
Ever had diabetes	√	√	√	√	√	Yes=1, No=0
Ever had cancer	√	√	√	√	√	Yes=1, No=0
Ever had lung disease	√	√	√		√	Yes=1, No=0
Ever had respiratory illness, such as asthma or emphysema		√		√	√	Yes=1, No=0
Ever had heart problems or heart attack	√	√	√	√	√	Yes=1, No=0
Ever had stroke	√	√	√	√	√	Yes=1, No=0
Ever had arthritis	√	√	√	√	√	Yes=1, No=0
Ever had anxiety, nervous or psychiatric problems	√	√	△		√	Yes=1, No=0
Ever had dyslipidemia or high cholesterol		△	√		√	Yes=1, No=0
Ever had Parkinson disease		√	√			Yes=1, No=0
Ever had cataracts		√	√			Yes=1, No=0

Item	Whether used in this study					Threshold
	HRS	ELSA	SHARE	MHAS	CHARLS	
	Wave 7-16	Wave 1-2, 4-6	Wave 1-2, 4-9	Wave 1-6	Wave 1-4	
Ever had hip fracture		√	√			Yes=1, No=0
Ever had stomach/digestive disease (e.g., stomach or duodenal ulcer, or peptic ulcer)			√		√	Yes=1, No=0
Ever had liver disease					√	Yes=1, No=0
Ever had kidney disease					√	Yes=1, No=0
Whether experiences frequent pain	△	√		√		Yes=1, No=0
Had back problems	√					Yes=1, No=0
BADL: Any difficulty in walking across room	√	√	√	√		Yes=1, No=0
BADL: Any difficulty in dressing	√	√	√	√	√	Yes=1, No=0
BADL: Any difficulty in bathing (or showering)	√	√	√	√	√	Yes=1, No=0
BADL: Any difficulty in eating	√	√	√	√	√	Yes=1, No=0
BADL: Any difficulty in getting in/out bed	√	√	√	√	√	Yes=1, No=0
BADL: Any difficulty in using the toilet	√	√	√	√	√	Yes=1, No=0
BADL: Any difficulty in controlling urination and defecation					√	Yes=1, No=0
IADL: Any difficulty in using telephone	√	√	√		△	Yes=1, No=0
IADL: Any difficulty in managing money	√	√	√	√	√	Yes=1, No=0
IADL: Any difficulty in taking medications	√	√	√	√	√	Yes=1, No=0
IADL: Any difficulty in shopping	√	√	√	√	√	Yes=1, No=0
IADL: Any difficulty in preparing hot meal	√	√	√	√	√	Yes=1, No=0
IADL: Any difficulty in using a map	△	√	√			Yes=1, No=0
IADL: Any difficulty in doing work around the house and garden		√	√		√	Yes=1, No=0
Mobility: Any difficulty in walking several blocks	√			√		Yes=1, No=0
Mobility: Any difficulty in walking 100 meters/yards		√	√		√	Yes=1, No=0
Mobility: Any difficulty in sitting for about 2 hours	√	√	√	√		Yes=1, No=0
Mobility: Any difficulty in jogging one mile or kilometre	√			√	√	Yes=1, No=0
Mobility: Any difficulty in walking one block or one kilometre	√			√	√	Yes=1, No=0

Item	Whether used in this study					Threshold
	HRS	ELSA	SHARE	MHAS	CHARLS	
	Wave 7-16	Wave 1-2, 4-6	Wave 1-2, 4-9	Wave 1-6	Wave 1-4	
Mobility: Any difficulty in getting up from a chair after sitting for long periods	√	√	√	√	√	Yes=1, No=0
Mobility: Any difficulty in climbing one flight of stairs without resting	√	√	√	√		Yes=1, No=0
Mobility: Any difficulty in climbing several flights of stairs without resting	√	√	√	√	√	Yes=1, No=0
Mobility: Any difficulty in stooping, kneeling, or crouching	√	√	√	√	√	Yes=1, No=0
Mobility: Any difficulty in lifting or carrying weights over 10 pounds or 5 kilos	√	√	√	√	√	Yes=1, No=0
Mobility: Any difficulty in picking up a dime/coin from the table	√	√	√	√	√	Yes=1, No=0
Mobility: Any difficulty in reaching arms above shoulder level	√	√	√	√	√	Yes=1, No=0
Mobility: Any difficulty in pushing or pulling large objects	√	√	√	√		Yes=1, No=0

Check marks (√) represent the items were fully used in all waves of a cohort. Tiny triangles (△) indicate items partially used across cohort waves due to missing data in two or fewer waves. Blanks represent the items were not used in a cohort. Information on conditions of self-rated memory, pain, and any difficulty in using a map were utilized in wave 7 to wave 15 in HRS; conditions of self-rated memory were used in wave 1, 2, and 4, underweight and obesity based on BMI were used in wave 2, 4, and 6, whether ever had dyslipidemia or high cholesterol were used in wave 2, 4 to 6 in ELSA; conditions of self-rated memory were used in wave 4 to 9, psychiatric problems were used in wave 2, 4 to 9 in SHARE; conditions of self-rated memory were used in wave 3 to 6 in MHAS; conditions of unweight and obesity were used in wave 1 to 3, any difficulty in using telepho ne were used in wave 2 to 4 in CHARLS.

Table S6. Distribution of frailty index across cohorts at baseline

Cohort	N	Mean	SD	Min	p1	p5	p10	p25	Median	p75	p90	p95	p99	Max
HRS	29398	0.2159	0.169	0	0.0125	0.0312	0.0469	0.0875	0.1656	0.3031	0.4679	0.5656	0.7361	0.9062
ELSA	12220	0.1425	0.1341	0	0	0.0122	0.0213	0.0483	0.0952	0.1951	0.3476	0.4421	0.5732	0.8079
SHARE	104132	0.1372	0.1226	0	0.0042	0.0208	0.0312	0.0542	0.0972	0.1786	0.2979	0.3948	0.6052	0.8838
MHAS	21017	0.1744	0.1445	0	0.0147	0.0303	0.0391	0.0675	0.1254	0.2426	0.3819	0.4708	0.6579	0.8872
CHARLS	13547	0.1621	0.1288	0	0.0086	0.023	0.0356	0.0694	0.1284	0.2185	0.3333	0.4321	0.6062	0.8125

Abbreviations: N: sample size; SD: standard deviation; p1: 1st percentile; p5: 5th percentile; p10: 10th percentile; p25: 25th percentile; p75: 75th percentile; p90: 90th percentile; p95: 95th percentile; p99: 99th percentile.

Table S7. Interaction results based on likelihood ratio tests

Class	-2LL in Model 1 without interaction items	-2LL in Model 2 with interaction items	Δ(-2LL)	df	*P for interaction*
**Individual level**					
Gender	1065097.48	1064971.60	125.88	2	4.629×10^{-28}
Educational attainment	1056406.44	1056117.54	288.9	4	2.685×10^{-61}
Marital status	1069920.80	1069807.24	113.56	2	2.192×10^{-25}
Current smoking	1066809.82	1066660.92	148.9	2	4.643×10^{-33}
**Country level**					
GDP per capita	1053777.72	1053567.46	210.26	2	2.201×10^{-46}
Gini index	1068478.49	1067318.59	1159.9	2	1.35×10^{-252}
Current health expenditure	1058103.79	1057855.73	248.06	2	1.363×10^{-54}
Tax revenue	1066147.59	1064979.54	1168.05	2	2.30×10^{-254}
Civil society participation index	1055097.67	1054926.92	170.75	2	8.358×10^{-38}
Government effectiveness	1053801.02	1053598.10	202.92	2	8.639×10^{-45}
Control of corruption	1057509.49	1057336.81	172.68	2	3.184×10^{-38}
Political stability and absence of violence/terrorism	1068214.71	1066945.31	1269.4	2	2.26×10^{-276}

Abbreviation: -2LL: -2 Log-likelihood; df: degrees of freedom difference.

Table S8. Basic characteristics of included and excluded participants

	Inclusion	Exclusion	Exclusion (1)	Exclusion (2)	Exclusion (3)	Exclusion (4)
Overall						
Size	241517	61203	6491	52272	2220	220
Age	63.99 (10.09)	63.14 (9.97)	63.74 (10.01)	62.98 (9.94)	64.25 (10.06)	72.39 (10.47)
Gender						
Women	130268 (53.94)	32657 (53.36)	3556 (54.78)	27871 (53.32)	1,140 (51.35)	90 (40.91)
Men	111249 (46.06)	28546 (46.64)	2935 (45.22)	24401 (46.68)	1,080 (48.65)	130 (59.09)
HRS						
Size	30786	1388	26	1149	208	5
Age	63.84 (10.94)	59.38 (9.52)	56.96 (8.06)	57.91 (8.58)	67.30 (10.10)	79.60 (12.99)
Gender						
Women	17091 (55.52)	697 (50.22)	17 (65.38)	567 (49.35)	109 (52.40)	4 (80.00)
Men	13695 (44.48)	691 (49.78)	9 (34.62)	582 (50.65)	99 (47.60)	1 (20.00)
ELSA						
Size	16694	4474	318	3025	1119	12
Age	63.51 (9.85)	60.75 (9.12)	55.07 (5.48)	59.73 (8.60)	65.03 (9.60)	70.92 (9.85)
Gender						
Women	8886 (53.23)	2491 (55.68)	162 (50.94)	1560 (51.57)	766 (68.45)	3 (25.00)
Men	7808 (46.77)	1983 (44.32)	156 (49.06)	1465 (48.43)	353 (31.55)	9 (75.00)
SHARE						
Size	153307	49175	5470	43361	212	132
Age	64.81 (10.11)	63.79 (9.99)	64.80 (9.98)	63.60 (9.94)	69.47 (11.89)	74.61 (9.78)
Gender						
Women	83448 (54.43)	26751 (54.40)	3057 (55.89)	23502 (54.20)	137 (64.62)	55 (41.67)
Men	69859 (45.57)	22424 (45.60)	2413 (44.11)	19859 (45.80)	75 (35.38)	77 (58.33)
MHAS						
Size	23822	2805	301	2200	236	68
Age	60.29 (8.87)	59.67 (9.81)	58.92 (8.77)	59.66 (10.02)	58.30 (7.46)	68.10 (10.45)
Gender						
Women	12430 (52.18)	1273 (45.38)	126 (41.86)	1030 (46.82)	91 (38.56)	26 (38.24)
Men	11392 (47.82)	1532 (54.62)	175 (58.14)	1170 (53.18)	145 (61.44)	42 (61.76)
CHARLS						
Size	16908	3361	376	2537	445	3
Age	62.41 (8.70)	61.41 (9.54)	60.03 (8.73)	61.59 (9.69)	61.56 (9.22)	65.33 (7.09)
Gender						
Women	8413 (49.76)	1445 (42.99)	194 (51.60)	1212 (47.77)	37 (8.31)	2 (66.67)
Men	8495 (50.24)	1916 (57.01)	182 (48.40)	1325 (52.23)	408 (91.69)	1 (33.33)

Inclusion: individuals aged 50 and above who enrolled in the cohorts.

Exclusion: Exclusion (1) + Exclusion (2) + Exclusion (3) + Exclusion (4).

Exclusion (1): individuals with missing records on frailty across all waves while alive.

Exclusion (2): individuals with missing or single-wave records for health and work status across all waves.

Exclusion (3): individuals with missing subpopulation identifiers.

Exclusion (4): individuals with recorded death dates prior to the last follow-up date wrongly.

Table S9. Health working related life expectancy at age 50 years by frailty

Frailty status	TLE			HnWLE			**HWLE**			nHnWLE			nHWLE		
	Years	LCI	UCI	Years	LCI	UCI	**Years**	**LCI**	**UCI**	Years	LCI	UCI	Years	LCI	UCI
Robust	35.56	35.33	35.79	14.14	13.95	14.34	**11.87**	**11.74**	**12.00**	6.08	5.96	6.20	3.46	3.37	3.55
Prefrail	32.76	32.59	32.94	10.04	9.91	10.17	**7.35**	**7.19**	**7.50**	10.30	10.17	10.44	5.07	4.95	5.20
Frail	25.92	25.73	26.11	4.96	4.84	5.08	**3.77**	**3.57**	**3.97**	12.29	12.09	12.48	4.91	4.74	5.08

Abbreviations: LCI: lower 95% CI; UCI: upper 95% CI.

Table S10. Imputed values for missing information on country-level factors through differential imputed methods

Country	Factor	Year	M1	M2	M3	M4	M5
Hungary	Gini index	2018	31.033	30.600	30.600	30.600	30.600
Hungary	Gini index	2019	31.350	30.600	30.600	30.600	30.600
Mexico	Gini index	2011	49.015	48.650	48.299	48.650	47.700
Mexico	Gini index	2017	46.550	46.450	46.401	46.450	46.900
Mexico	Tax revenue	2002	9.244	9.244	10.396	9.485	9.132
Netherlands	Gini index	2003	29.375	29.460	30.392	29.692	28.100
Spain	Tax revenue	2003	14.684	14.684	13.982	14.509	15.118
Switzerland	Gini index	2003	31.650	31.650	31.289	31.643	31.700
Switzerland	Gini index	2005	32.750	32.750	32.727	32.750	31.600
United Kingdom	Control of Corruption	2001	2.045	2.038	2.036	2.038	2.072
United Kingdom	Government Effectiveness	2001	1.785	1.774	1.787	1.774	1.769
United Kingdom	PSAVT	2001	0.781	0.853	0.950	0.902	1.033

M1 represents the method using the structural time series models on the basis of Kalman smoothing. For M2 to M4, missing data were imputed using interpolation for intermittent missing values, while the last observation carried forward (LOCF) method was applied for missing values at the end of the observation period. Specifically, M2, M3, and M4 used the linear, spline, and Stineman interpolation respectively. M5 represents the LOCF for all the missing values.

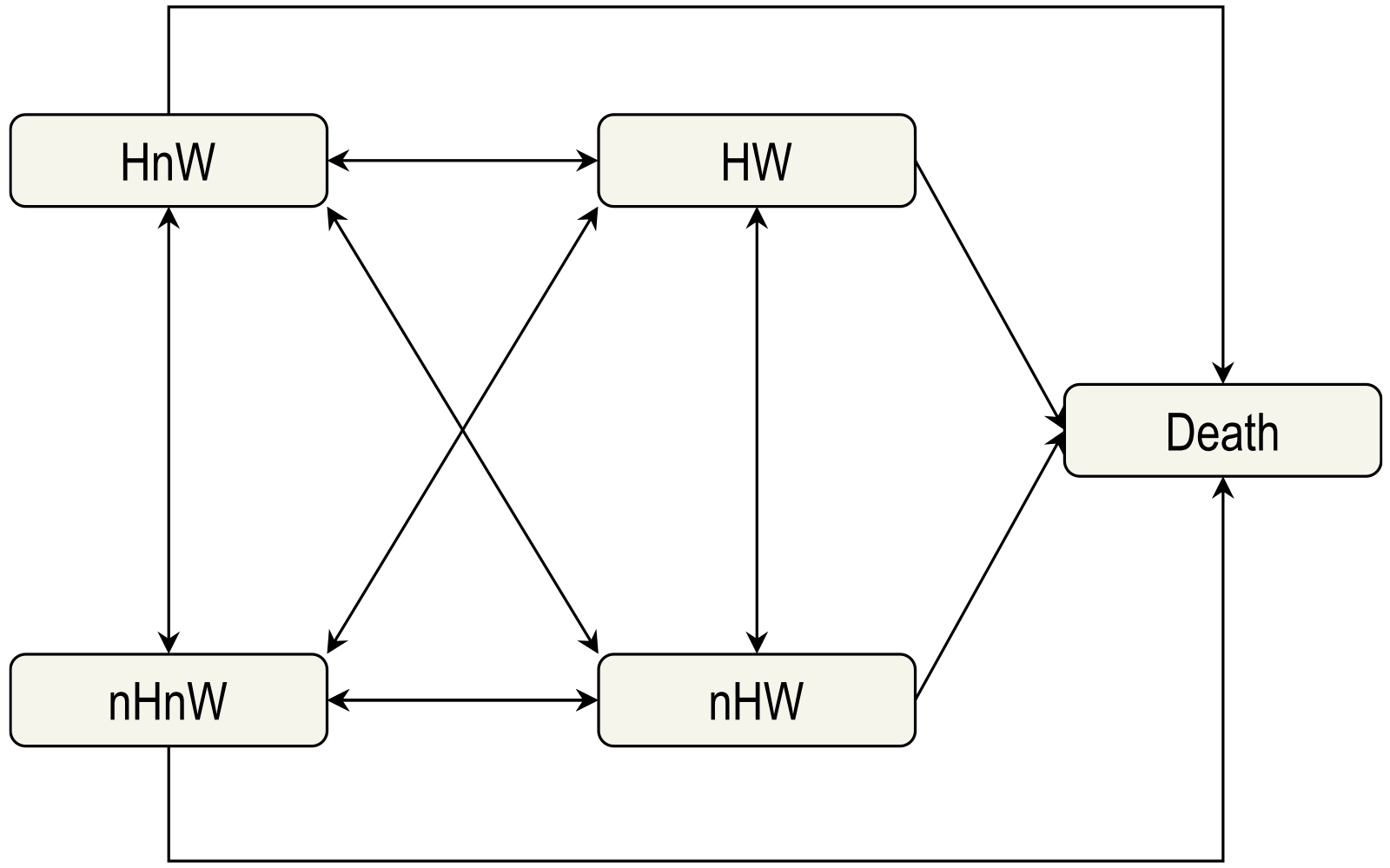

Figure S1. Transitions among five healthy working related states

HnW: healthy and not working. HW: healthy and working. nHnW: not healthy and not working. nHW: not healthy and working.

These abbreviations also apply to **Figure S2**.

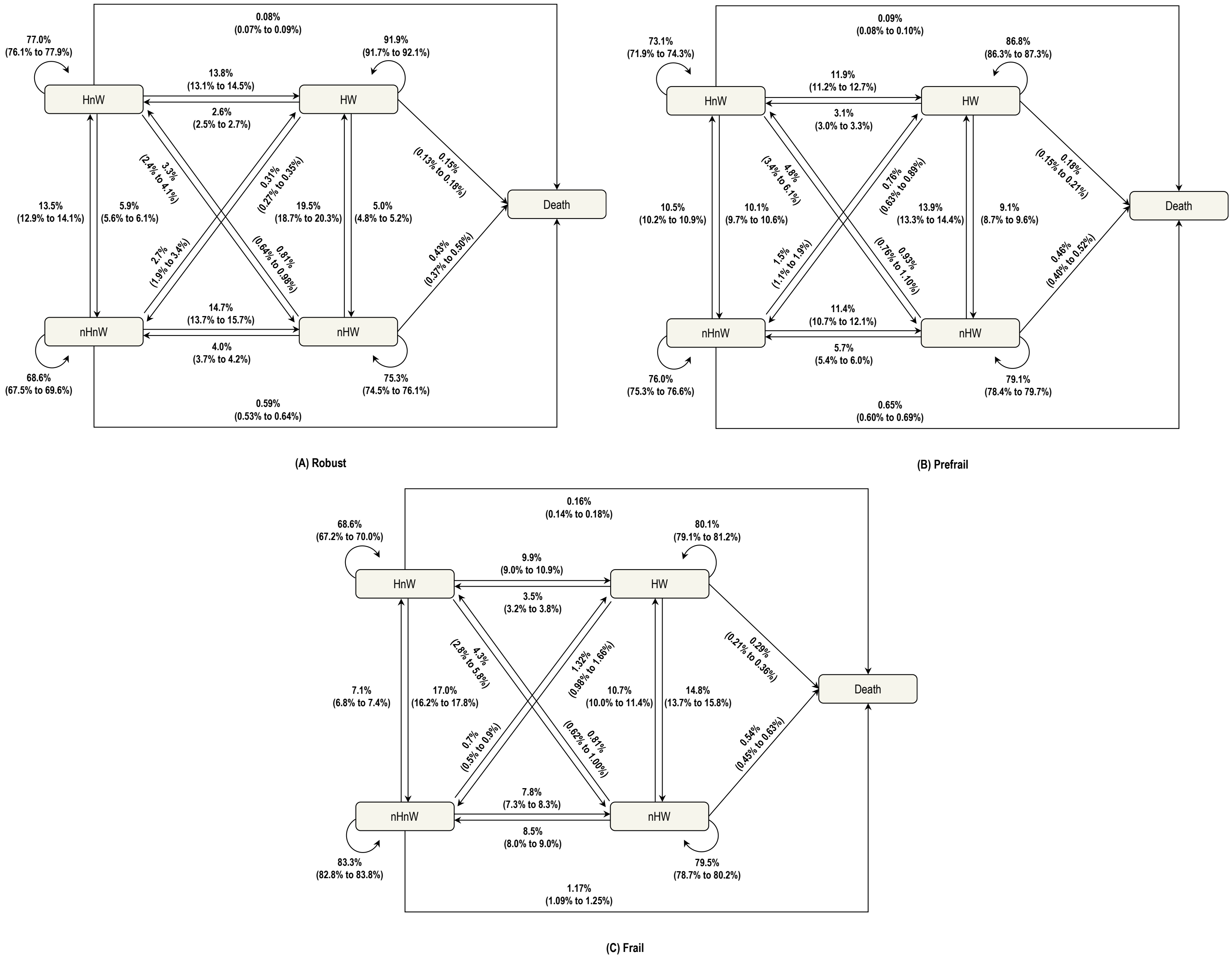

Figure S2. Estimated annual transition probabilities among five healthy working related states by frailty status at age 50 years

Values were presented as transition probabilities, with 95% CIs in parentheses.

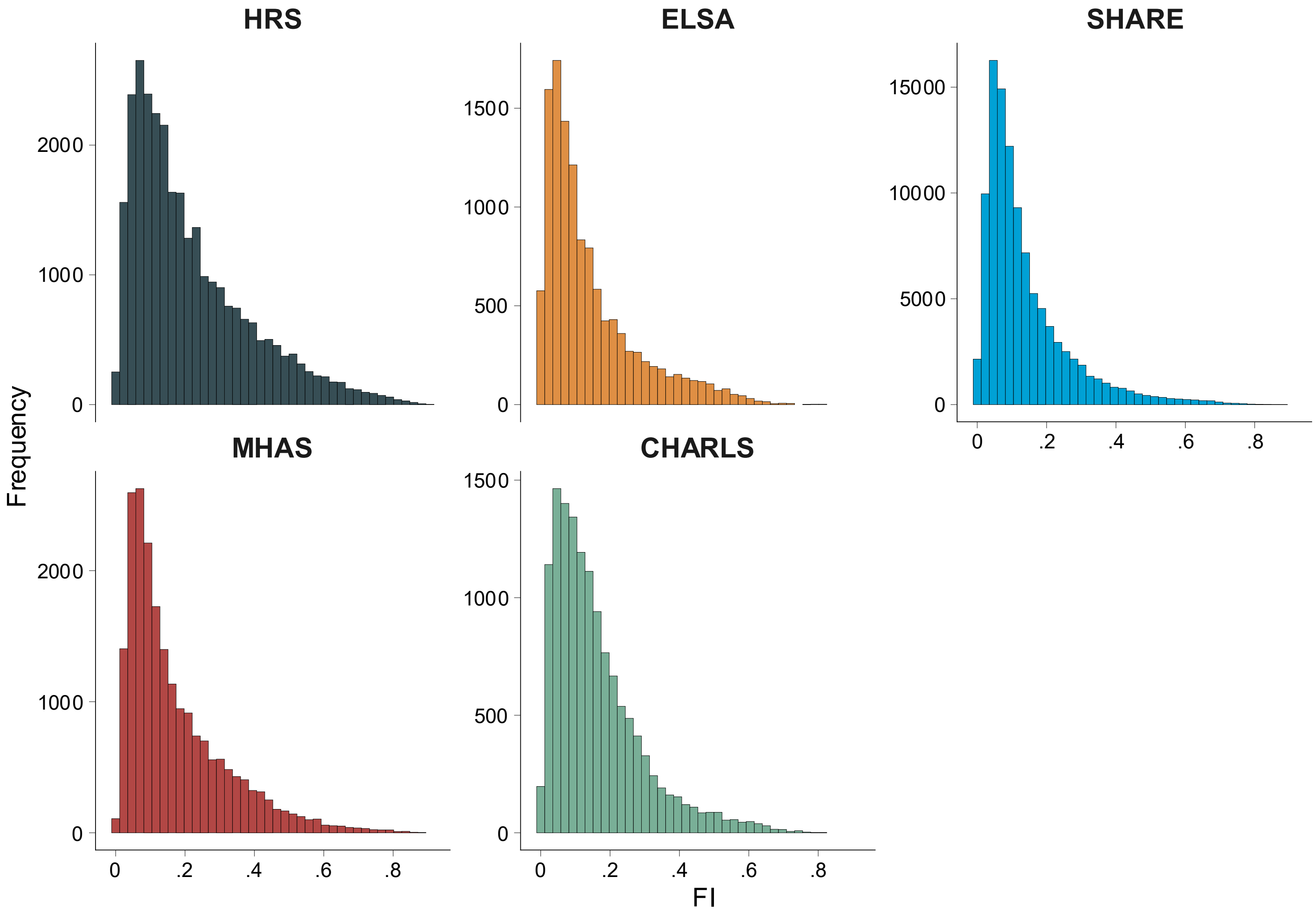

Figure S3. Distribution of frailty index across cohorts at baseline

FI – Frailty index.

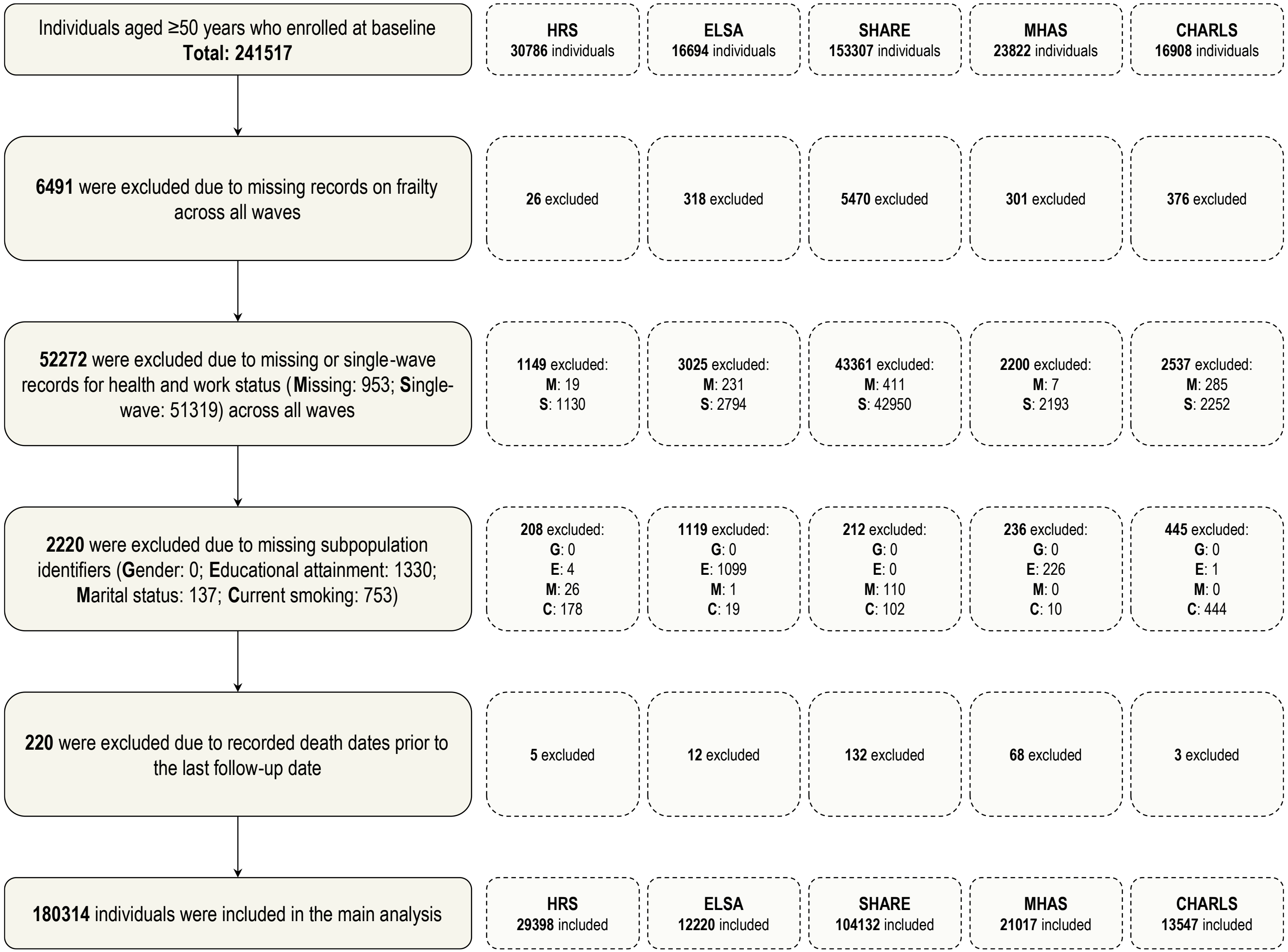

Figure S4. Process of sample selection

Inclusion criteria: individuals aged 50 and above who enrolled in the cohorts. Exclusion criteria: (1) individuals with missi ng records on frailty across all waves while alive; (2) individuals with missing or single-wave records for health and work status across all waves; (3) individuals with missing subpopulation identifiers; (4) individuals with recorded death dates prior to the last follow-up date wrongly.

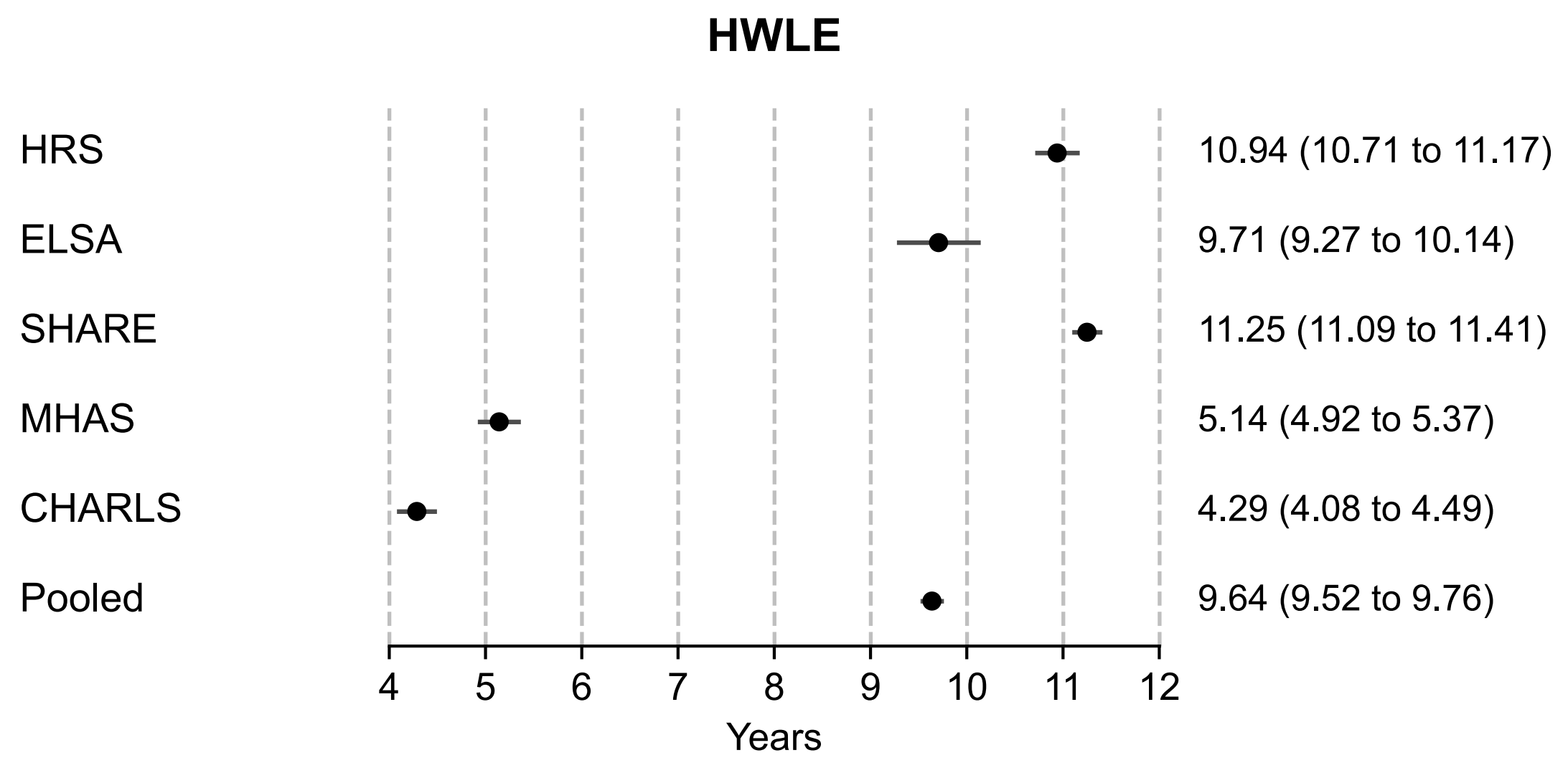

Figure S5. HWLE at age 50 years across cohorts

95% CIs for HWLE in parentheses.

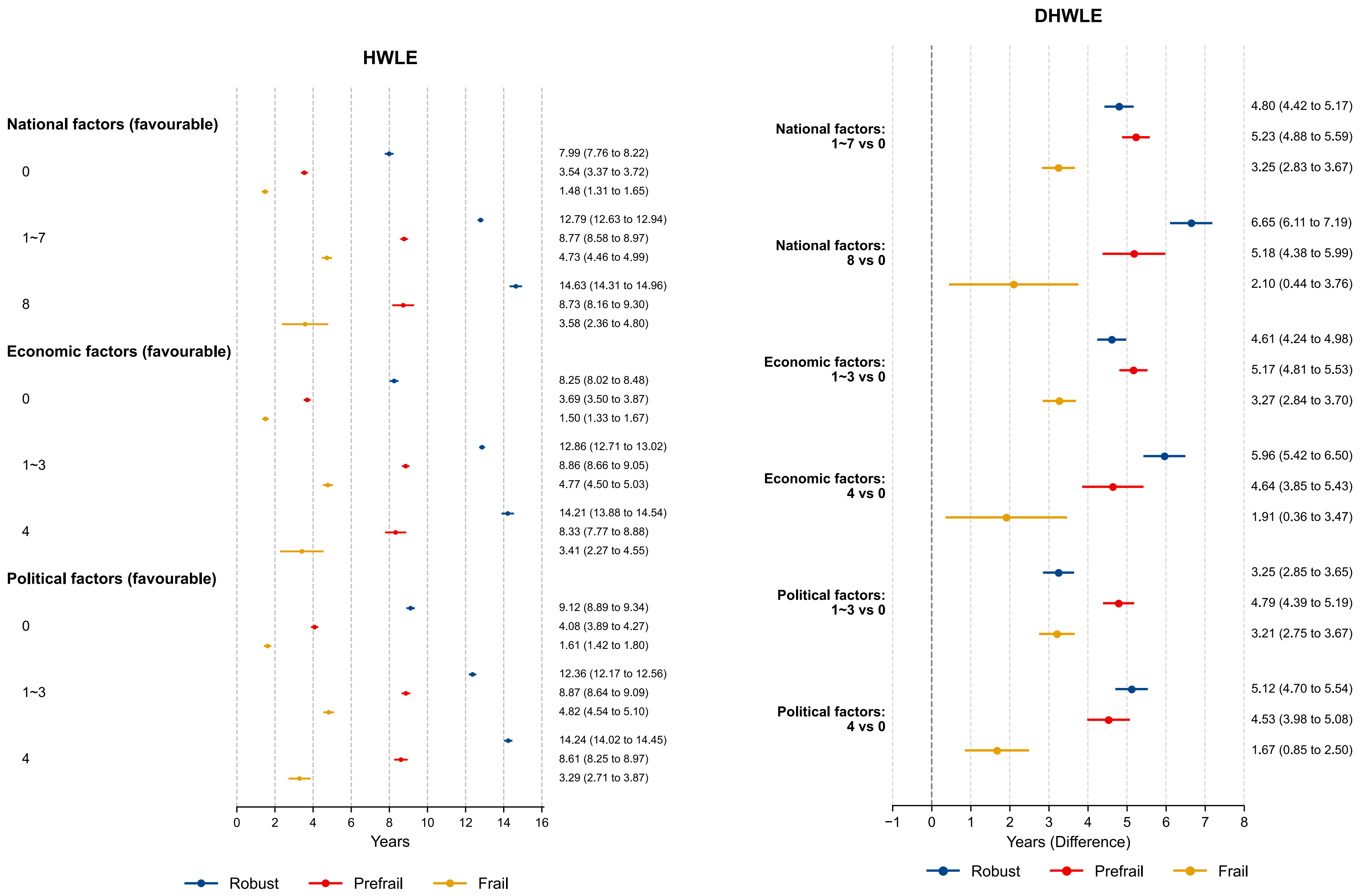

Figure S6. HWLE and DHWLE at age 50 years by frailty and numbers of favourable country-level indicators

95% CIs for HWLE and Bonferroni adjusted CIs for DHWLE in parentheses.

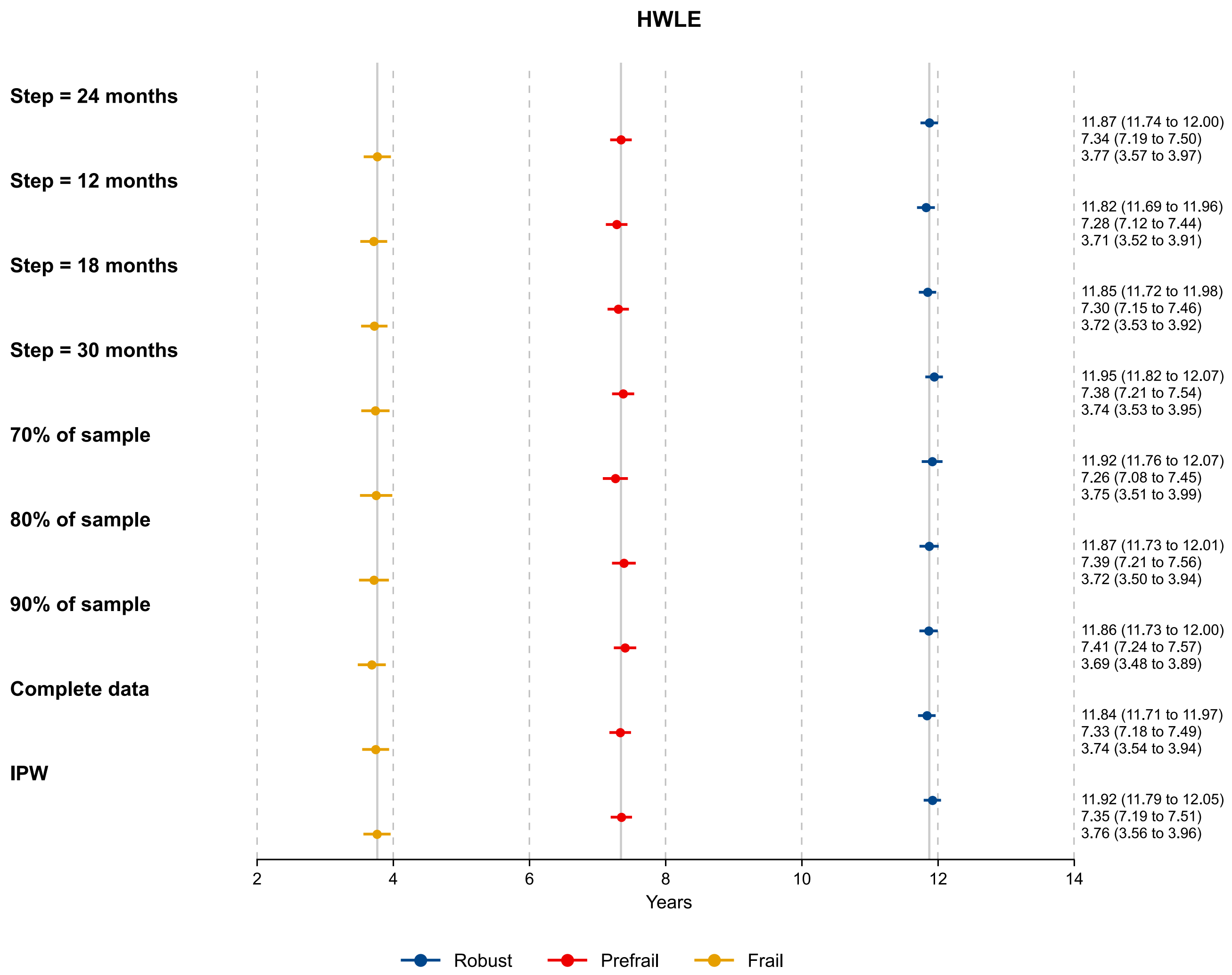

Figure S7. Comparison of HWLE by frailty through the differential methods

95% CIs in parentheses. The three solid grey vertical lines represent the point estimates of HWLE for each frailty status, calculated using a 24-month step size. The number of participants in the **complete data** is 182532.

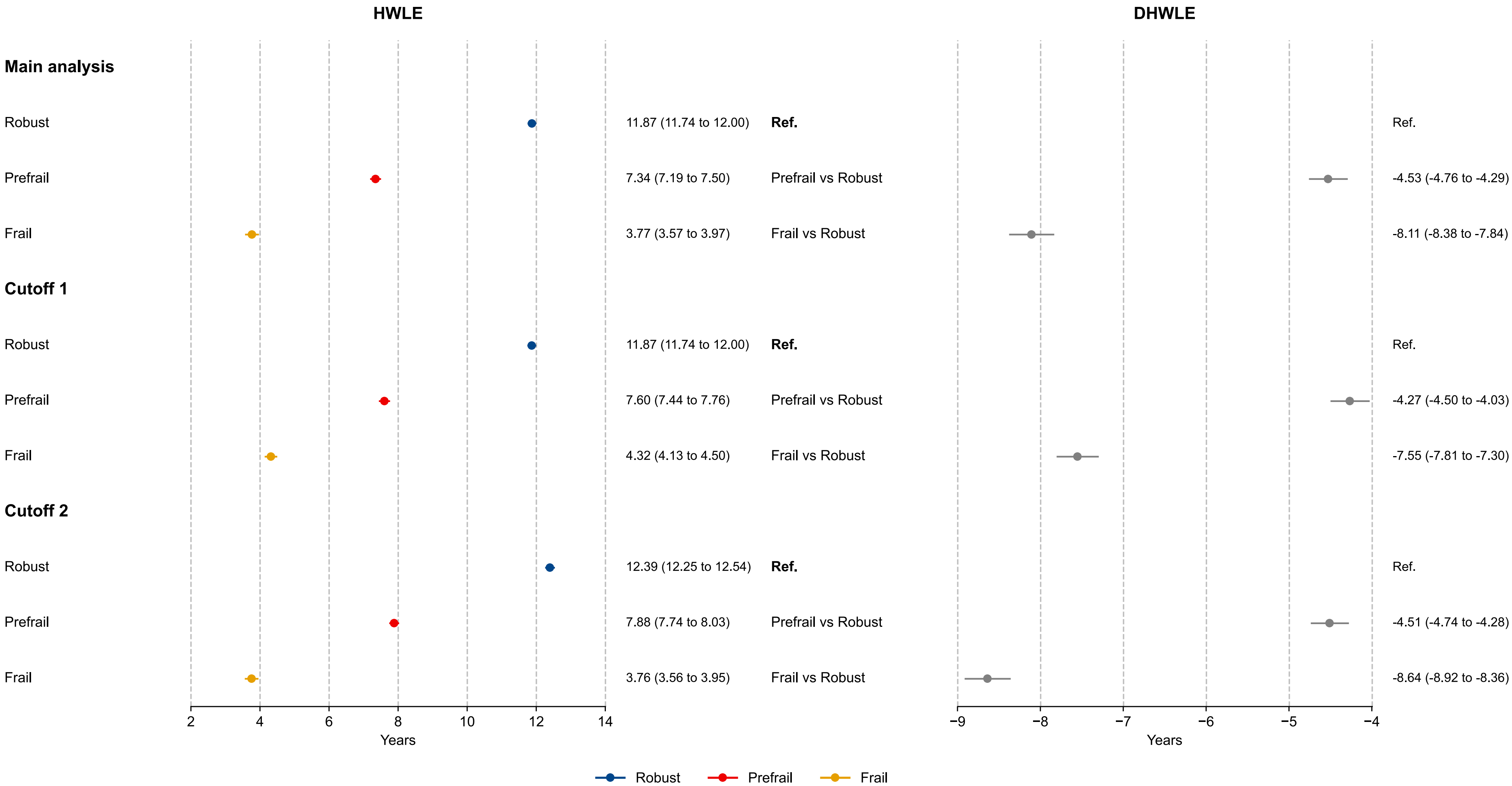

Figure S8. Comparison of HWLE by frailty under the differential cutoffs of frailty status

95% CIs for HWLE and Bonferroni adjusted CIs for DHWLE in parentheses. Cutoff 1 on frailty: robust (FI score < 0.08), prefrail (FI score ≥ 0.08 to < 0.25), and frail (FI score ≥ 0.25). Cutoff 2: robust (FI score ≤ 0.10), prefrail (FI score > 0.10 to < 0.21), and frail (FI score ≥ 0.21).

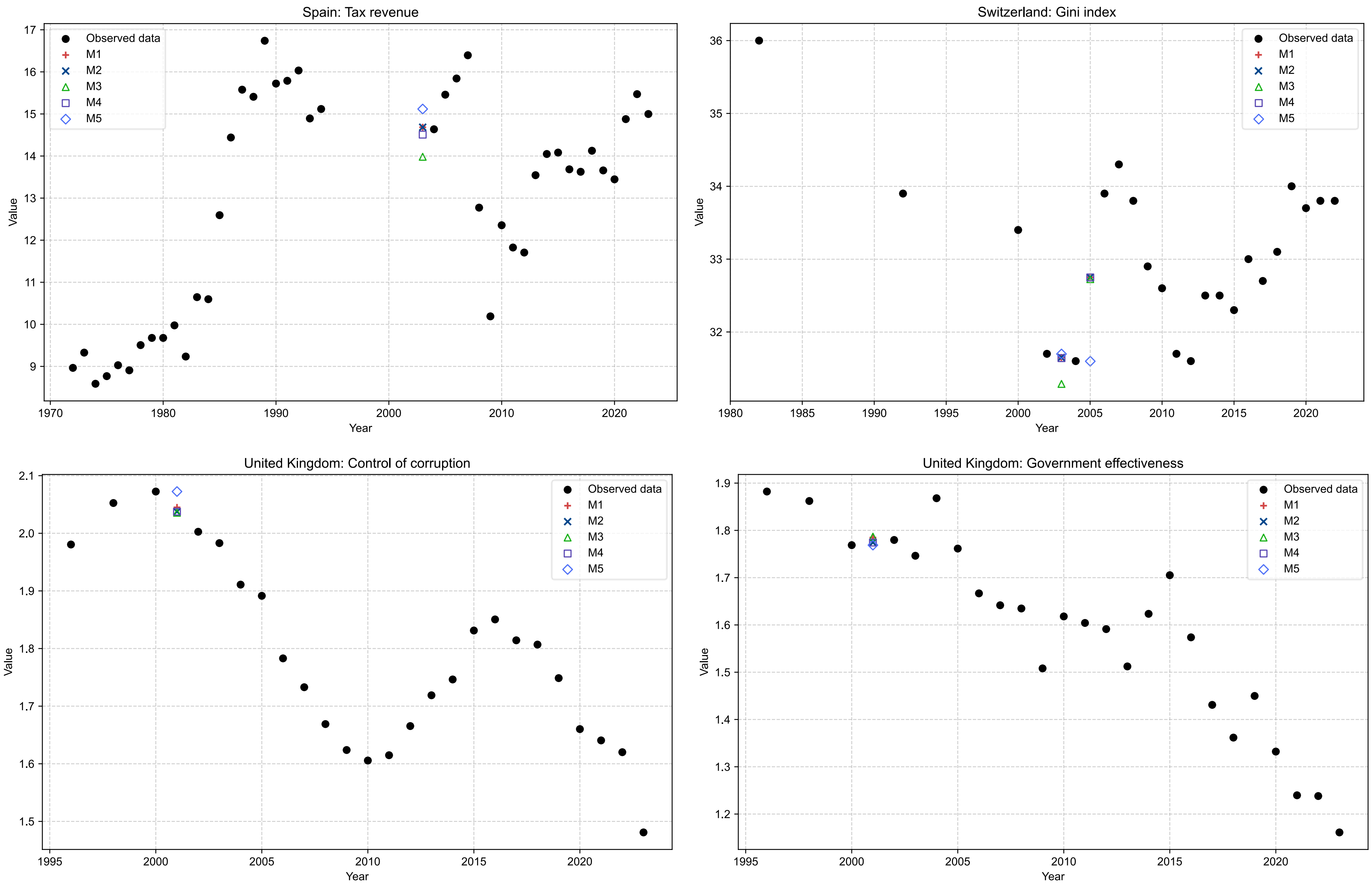
Spain: Tax revenue
Switzerland: Gini index
United Kingdom: Control of corruption
United Kingdom: Government effectiveness
Observed data
M1
M2
M3
M4
M5
Value
Year

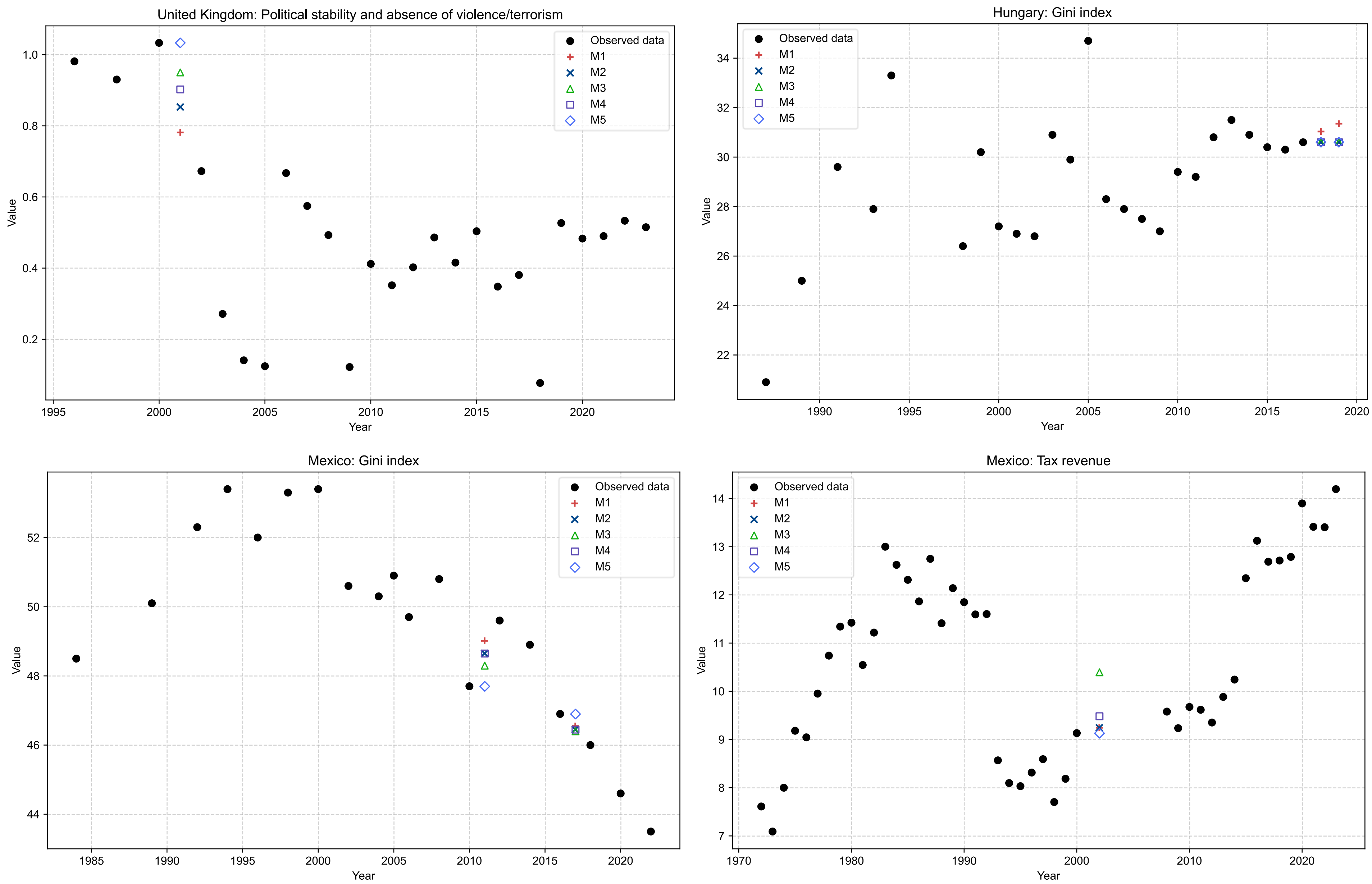
United Kingdom: Political stability and absence of violence/terrorism
Hungary: Gini index
Mexico: Gini index
Mexico: Tax revenue
Observed data
M1
M2
M3
M4
M5
Value
Year

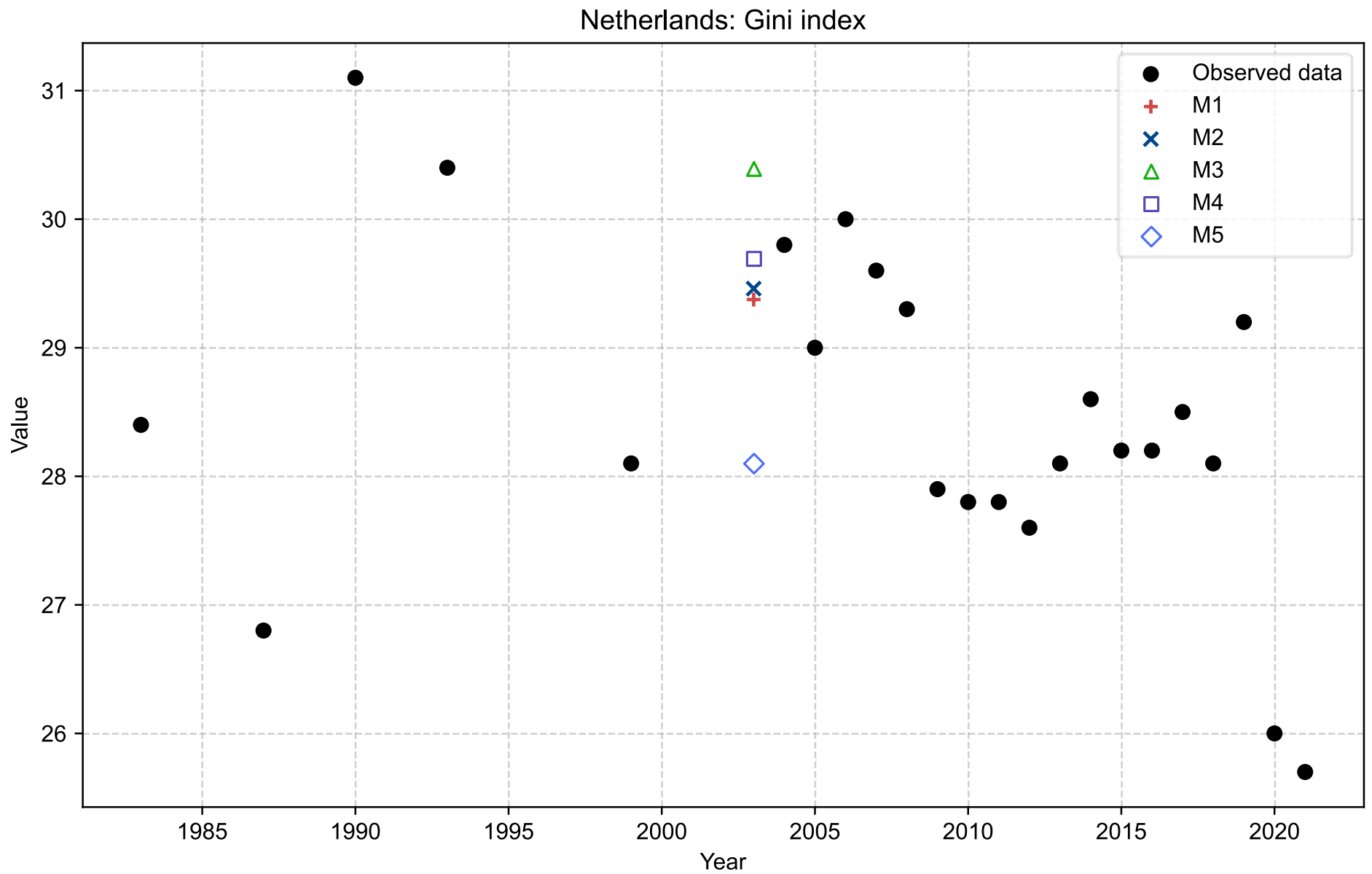

Figure S9. Comparison of the imputed values through alternative imputation methods

M1 represents the method using the structural time series models on the basis of Kalman smoothing. For M2 to M4, missing data were imputed using interpolation for intermittent missing values, while the last observation carried forward (LOCF) method was applied for missing values at the end of the observation period. Specifically, M2, M3, and M4 used the linear, spline, and Stineman interpolation respectively. M5 represents the LOCF for all the missing values.

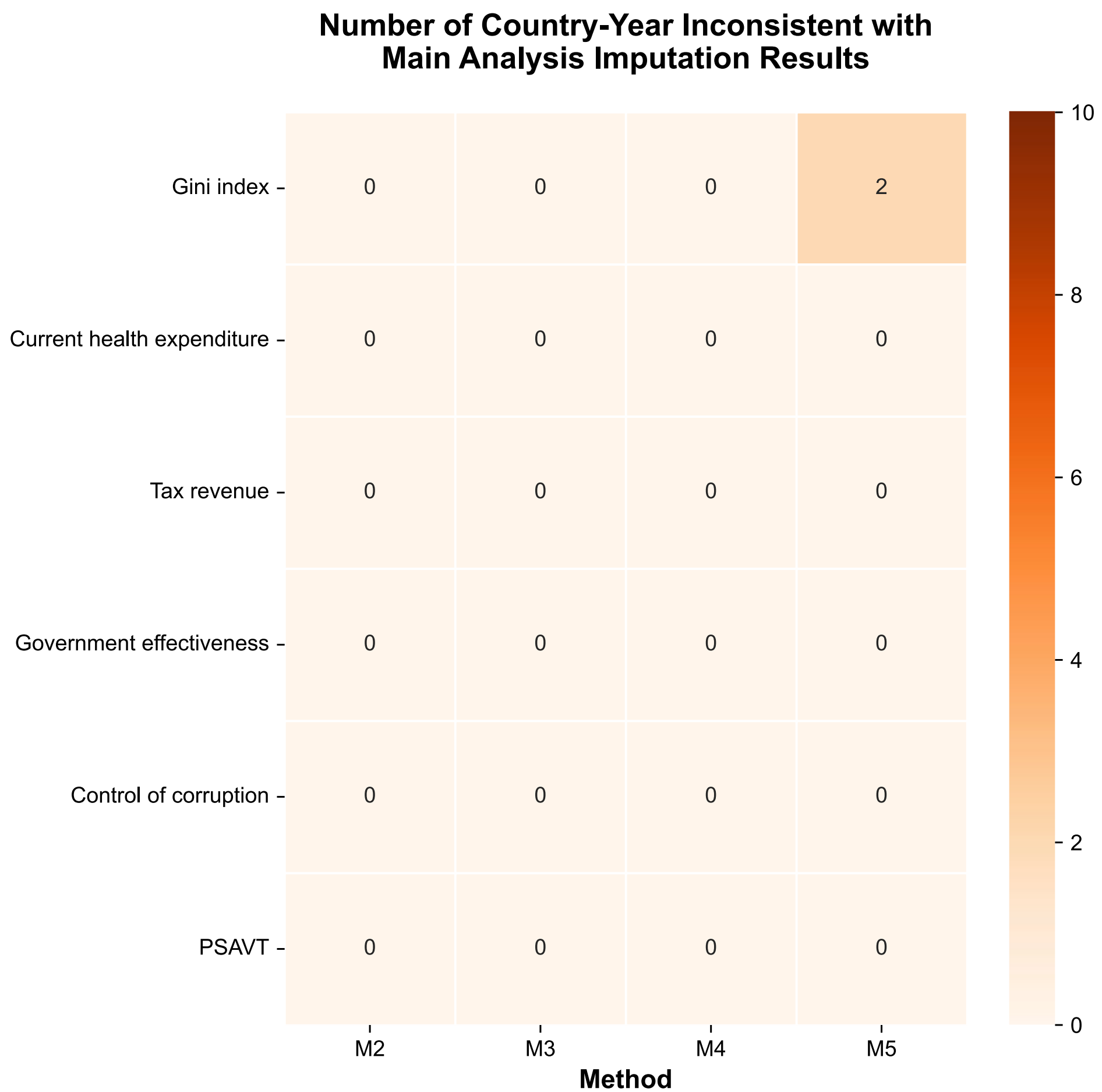

Figure S10. Number of country-year dichotomous information inconsistent with imputation results in the main analysis (M1)

M1 represents the method using the structural time series models on the basis of Kalman smoothing. For M2 to M4, missing data were imputed using interpolation for intermittent missing values, while the LOCF method was applied for missing values at the end of the observation period. Specifically, M2, M3, and M4 used the linear, spline, and Stineman interpolation respectively. M5 represents the LOCF for all the missing values.

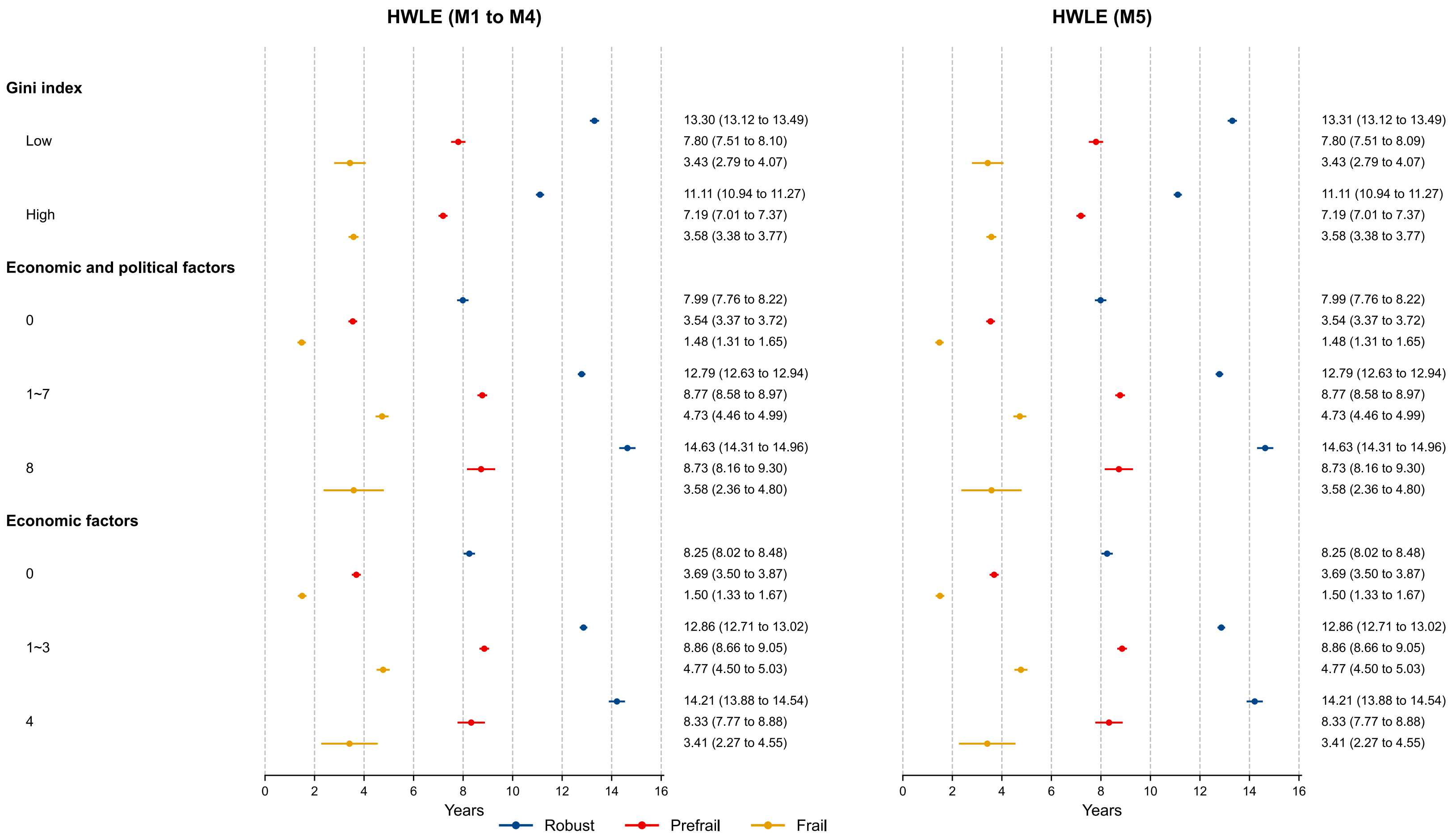

Figure S11. Comparison of HWLE by frailty through alternative imputation methods

M1 represents the method using the structural time series models on the basis of Kalman smoothing. For M2 to M4, missing data were imputed using interpolation for intermittent missing values, while the LOCF method was applied for missing values at the end of the observation period. Specifically, M2, M3, and M4 used the linear, spline, and Stineman interpolation respectively. M5 represents the LOCF for all the missing values.

When compared with the country-level binary variables obtained from M1 to M4, the dataset with missing values filled using M5 showed only two discrepancies (both in the Gini index). Consequently, comparisons of HWLE across frailty statuses were limited to stratifications based on the Gini index, the number of favourable macro- economic and political factors, and the number of favourable macro-economic factors.

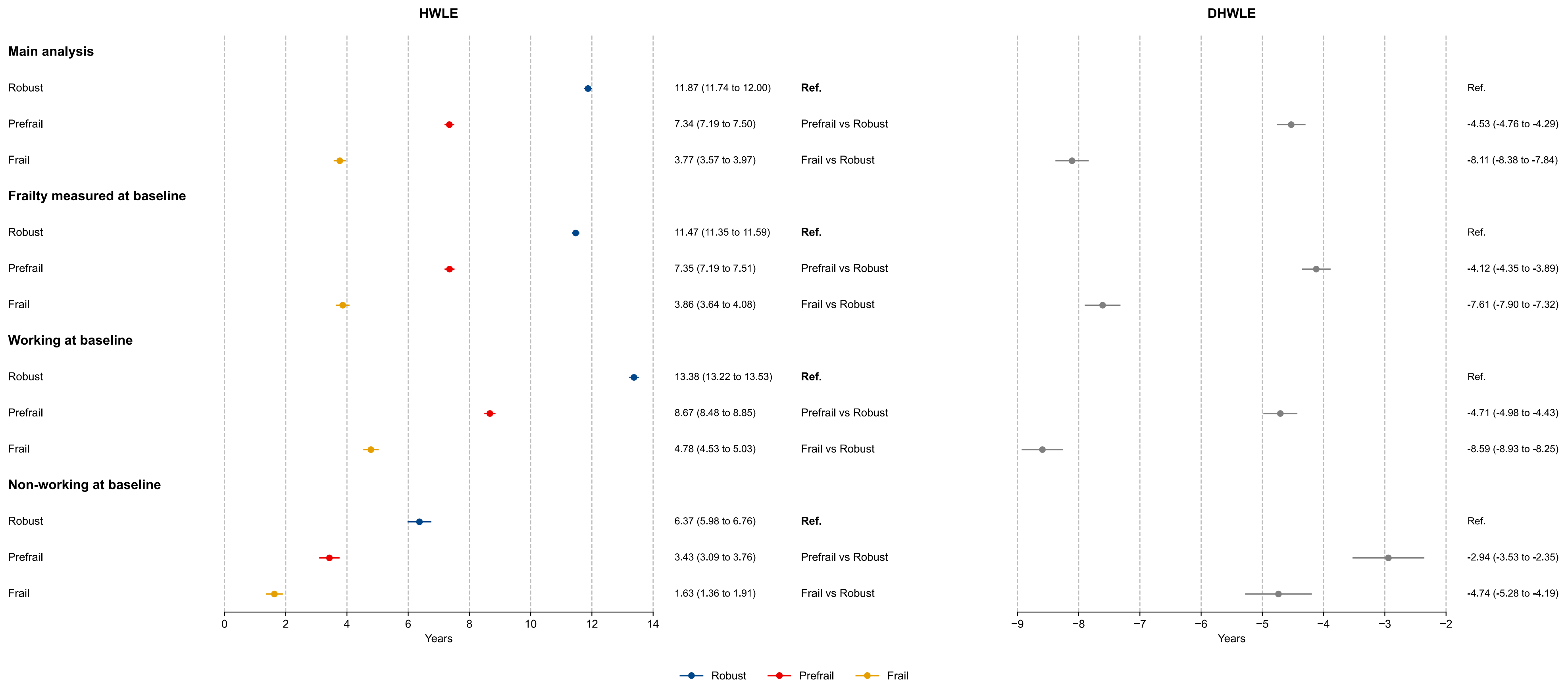

Figure S12. HWLE by baseline frailty status and stratified by baseline work state
